# Evaporation of a Reactive Nanofluid Sessile Drop: *Capturing Rapid Emergence of Surface Crystals with In Situ Synchrotron X‑ray Diffraction*

**DOI:** 10.1021/acs.langmuir.5c01989

**Published:** 2025-07-09

**Authors:** Patryk Wąsik, Anna Slastanova, Jacek M. Wąsik, Tim Snow, Alexander Gerrit de Bruin, Thomas Arnold, Wuge H. Briscoe

**Affiliations:** † National Synchrotron Light Source II, 8099 Brookhaven National Laboratory, Upton, New York 11973, United States; ‡ School of Chemistry, 1980 University of Bristol, Cantock’s Close, Bristol BS8 1TS, U.K.; § Bristol Centre for Functional Nanomaterials, HH Wills Physics Laboratory, University of Bristol, Tyndall Avenue, Bristol BS8 1TL, U.K.; ∥ School of Physics, HH Wills Physics Laboratory, Tyndall Avenue, Bristol BS8 1TL, U.K.; ⊥ 120796 Diamond Light Source, Diamond House, Harwell Science and Innovation Campus, Didcot, Oxfordshire OX11 0DE, U.K.

## Abstract

Mechanisms for surface pattern formation from evaporation of a *reactive* nanofluid sessile drop are not well understood. In contrast to the coffee-ring effect from inert particles, rapid chemical and morphological transformation of reactive nanoparticles upon rapid evaporative drying are challenging to probe experimentally. Here, using grazing-incidence X-ray surface scattering, the nanostructure of nascent surface patterns has been probed as a ZnO nanofluid sessile drop rapidly dries. The high temporal resolution enabled by the high flux of synchrotron X-rays allows the observation of the emergence of Zn­(OH)_2_ surface crystals from the onset of evaporation and their rapid evolution into the final residual surface pattern, *via* transient layered complexes evident from the temporary appearance of X-ray diffraction peaks preceding Zn­(OH)_2_ formation. The results offer mechanistic insights of morphogenesis of surface patterns from evaporation-induced self-assembly and self-organization of reactive nanofluids, previously untenable using other experimental methods.

## Graphical abstract

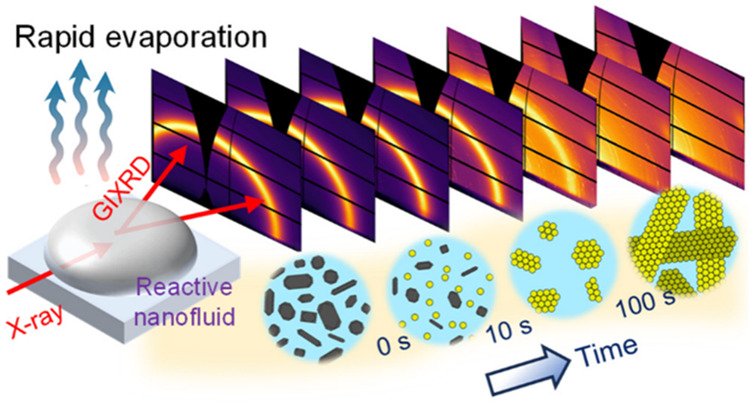

## Introduction

Drying of a particle laden droplet, widespread in everyday life and industrial processes, can lead to a plethora of residual surface patterns. The most common of these is the coffee-ring, for which the formation mechanism was first elucidated around two decades ago.^1–3^ The vast academic interest it stimulated continues to thrive today, and it is also directly relevant to producing functional devices and nanomaterials.^4–6^ In these previous studies, the dispersed particles were *inert*.

We have recently shown that, upon evaporation of a *reactive* ZnO nanofluid sessile drop, the residual pattern formation mechanism is very different from that associated with the coffee-ring, leading to the hierarchical surface pattern comprising solidified Bénard–Marangoni cells.^7,8^ The complex nature of the hierarchical morphologies in the residual surface patterns was descriptively identified as, e.g., being analogous to the foliage of red algae, Spanish dagger, or spider plant,^8^ and their geometric features can also be described more quantitatively *via* the fractal dimension analysis.^9^ The ultimate residual surface structure could also be tuned by the morphology and crystallinity of the dispersed ZnO particles^10^ and the substrate surface chemistry,^11^ as depicted in Figure 1. These findings have opened up new routes for fabrication of functional surface patterns *via* self-assembly induced by evaporation of reactive nanofluids.^12^ However, the mechanisms underpinning the residual surface pattern formation remain to be fully understood.

**Figure 1:**
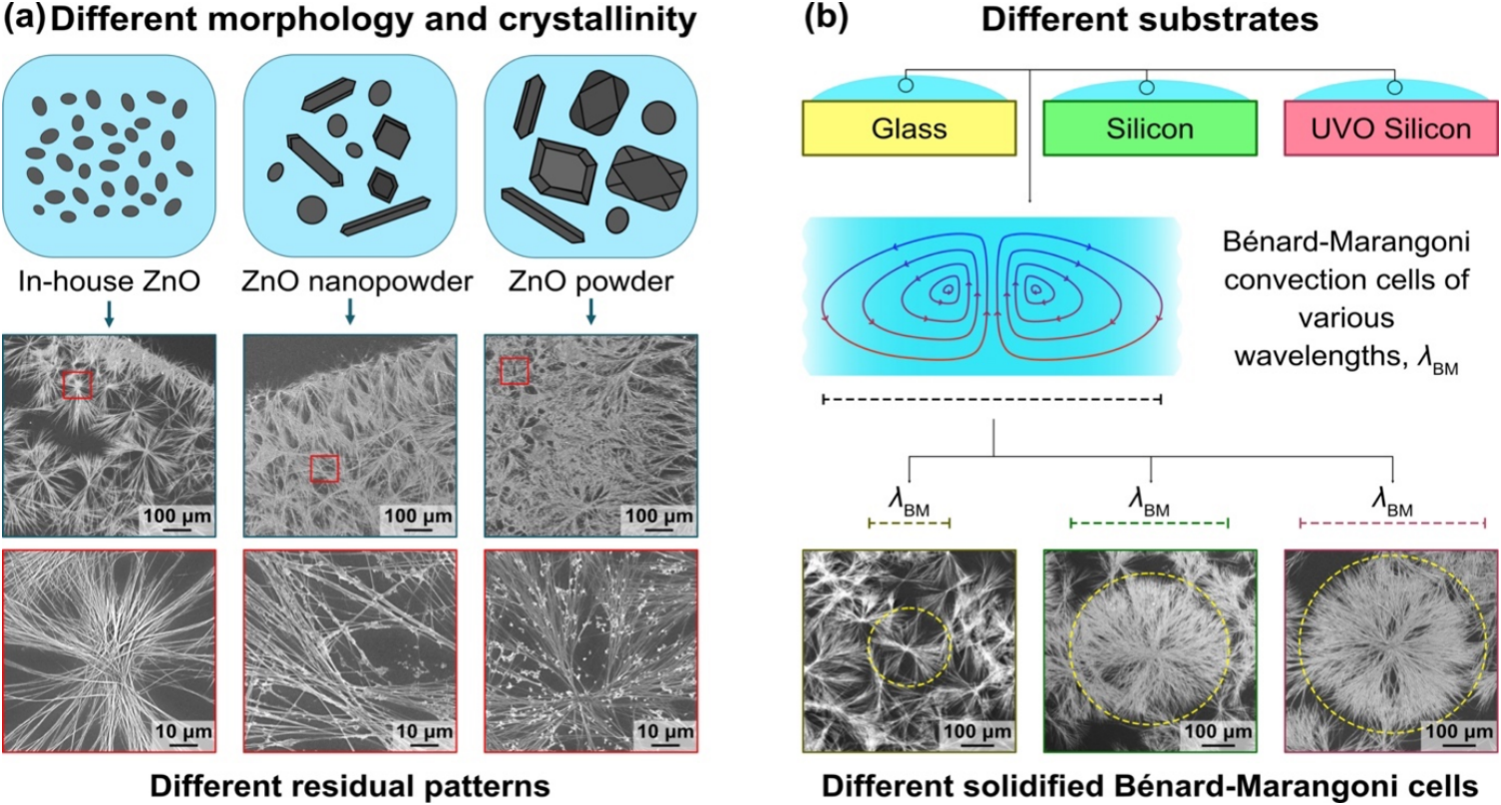
Rapid evaporative drying (∼200–300 s) of a sessile drop containing ZnO nanoparticles leads to fractal-like dendritic residual patterns, attributed to the Bénard–Marangoni instability triggered upon droplet thinning. The pattern morphology depends on (a) the ZnO particle crystallinity and (b) the substrate surface chemistry. The mechanism has been proposed to relate to rapid chemical and morphological transformation of reactive ZnO nanoparticles into Zn­(OH)_2_ molecular and nanocrystal species at the initial stage (a few to some tens of seconds) of the evaporation process, inferred from cryo-TEM imaging of the nanostructures suspended in the droplet collected at different time intervals, although no direct evidence has been provided. Some elements of this figure have been adapted with permission from previous work: ref 9, Copyright (2019) Elsevier, and ref 11, Copyright (2019) American Chemical Society.

The first attempt to explain the mechanism behind the formation of the hierarchical residual surface patterns from the evaporation of a sessile drop containing reactive ZnO nanorods dispersed in a mixture of isobutylamine and cyclohexane was made by Wu et al. in 2014.^7^ Central to the proposed mechanism is the hypothesis that the rapid chemical transformation of isobutylamine-coated ZnO nanorods into Zn­(OH)_2_ nanocrystals occurs at the early stage of evaporation. Subsequently, a combination of Marangoni and capillary flows, crystallization-mediated fingering instabilities at the dewetting front of the drying droplet, and hydrogen bonding between the *in situ* generated Zn­(OH)_2_ nanocrystals acts as the driving force for the assembly of nanocrystals into long fibers. Ambient moisture or water was found to play an important role in facilitating isobutylamine–ZnO transformation, and the detailed nanostructure and micromorphology in the residual thin film depend intricately on the ambient moisture. Other aspects of the evaporation-induced self-assembly process, such as controlling the morphology of the patterns *via* morphology and crystallinity of the ZnO particles and surface chemistry of the substrate as well as quantitative description of the geometric features of these BM cells, through the fractal dimension analysis, have been reported in refs 9–11

A revised mechanism proposed in 2018,^8^ again based on the hypothesis of rapid chemical transformation of nanoparticles at the onset of the evaporation process, has attempted to account for the macroscopic cellular patterns with dendritic micromorphologies formed within the central region of the pattern that is surrounded by a peripheral coffee-ring band. A detailed description of the proposed mechanisms is included in the Supporting Information (SI.01). Experimentally, the structural evolution of the surface pattern on nano/micro-/macroscopic levels was deduced using time-resolved transmission electron microscopy (TEM) and cryo-TEM observations of the constituent nanostructures (with ∼2 min temporal resolution) inside the droplet at different evaporation stages and video microscopy visualization of the capillary waves at the droplet surface, which manifested the Bénard–Marangoni instability in the thinning droplet. The driving force behind the Bénard–Marangoni cell formation was the viscosity gradient due to the solvent loss during evaporation, as discussed in refs 8,11 and SI.01. The hypothesis central to the proposed mechanism, i.e., the rapid ZnO → Zn­(OH)_2_ transformation on the surface, was not verified directly.

Different techniques have been previously used to study evaporation-induced surface patterns, including scanning electron microscopy (SEM)^13–15^ and TEM,^16,17^ and also their derivations such as cryogenic TEM^12,18^ or TEM tomography.^19,20^ Other methods include optical microscopy,^21–23^ polarized optical microscopy,^24,25^ confocal microscopy,^22,26–28^ optical spectroscopy,^24,29^ Raman scattering and spectroscopy,^17,26,30^ atomic force microscopy,^21,25,27,30,31^ and nuclear magnetic resonance spectroscopy.^32^ Dynamic behaviors of evaporative drying are more difficult to probe, and techniques such as liquid cell electron microscopy,^33–35^ contact angle measurements,^22,23,28,36^ dynamic light scattering,^29,37,38^ and neutron scattering^37–39^ can probe different aspects of the drying process with limited temporal solution.

The key mechanistic feature proposed for the evaporation of reactive ZnO nanofluids is the rapid nanoparticle morphological and chemical transformation at the initial drying stage (first tens of seconds upon evaporation),^8^ and such fast dynamics is inaccessible using the aforementioned experimental techniques. High brilliance synchrotron X-rays have been employed to study drying drops and thin films in real time, e.g., using grazing-incidence small-angle X-ray scattering (GISAXS) to monitor self-assembly of nanoparticles into superlattices,^40–47^ supramolecular materials into fibers,^48^ and polymers into membranes.^49^ Grazing-incidence wide-angle X-ray scattering (GIWAXS) has been used to probe crystallographic orientation evolution of nanocrystals in superlattices^50–52^ and wet-coated thin films.^53^ Hitherto, synchrotron X-ray scattering has not been applied to study dynamic surface pattern formation in the evaporation-induced self-assembly process from reactive nanofluids.

Here, we have utilized *in situ* grazing-incidence X-ray diffraction (GIXRD) to track the temporal evolution of the crystal structures as they form on the surface within a fast-drying sessile ZnO nanofluid drop. High temporal resolution allowed us to resolve fast intermediate steps in the surface pattern formation during the early stages of evaporation, which began immediately after droplet deposition and typically completed within 200–300 s. We studied three different types of ZnO nano/microparticles and different substrates, including glass slides, pristine and silanized silicon, and mica. Our results offer mechanistic insights into the morphogenesis of the surface patterns in the rapid evaporation process involving reactive nanofluids. The study also points to the feasibility of the GIXRD for studying other reactive nanofluids, such as CuO, CdO, and HgO or organic mixtures/reactive nanostructured surfaces, e.g., with ZnO nanorods, nanourchins, and nanopallets, which is the focus of our ongoing work.

## Experimental Section

### Particles

As previously reported, the dissolution or reactivity of ZnO particles depends on their crystallinity,^10^ which could be conveniently characterized by the coherence length, *L*_a_, of their diffraction peaks, with a higher value indicating a higher crystallinity and thus slower dissolution kinetics. Here, different particles were used for nano/microfluids preparation, with their average *L*_a_ values indicated as follows. These included the *in-house* synthesized ZnO nanoparticles (*ca* 10 nm in diameter; average *L*_a_ ∼12 ± 3 nm) and commercially acquired ZnO nanopowder (Sigma-Aldrich, < 100 nm particle size, ∼80% Zn basis; average *L*_a_ ∼60 ± 11 nm), and ZnO powder (Sigma-Aldrich, ACS reagent, ≥ 99.0% (KT); average *L*_a_ ∼57 ± 8 nm). Details on the synthesis of the in-house ZnO nanoparticles, characterization of the ZnO nano/powders, and coherence length calculations have been described in ref 10 SI.02, and SI.09.

### Nano/Microfluid Preparation

ZnO particles were added to a mixture of cyclohexane (Fisher Chemicals, assay 99%) and isobutylamine (Sigma-Aldrich, assay 99%), 5:1 volume:volume (v/v) ratio, in the designated amount to produce 1 mg/mL suspensions. The use of isobutylamine as a reactive solvent promotes ZnO dissolution *via* a moisture-assisted pathway (described in SI.01), while cyclohexane acts as a nonreactive carrier solvent. These were sonicated in an ultrasonic bath (Ultrawave, Model QS5, U.K.) for 0.5 – 1 h to form homogeneous dispersions just before the *in situ* drying experiments.

### Substrates

Different substrates of size 1 × 1 cm^2^ were used, including standard microscope glass slides (type 7101, 0.8 – 1.0 mm thick), silicon wafers (UniversityWafer Inc., ID 452, 100 mm diameter, P type, B dopant, < 100>, 0–100 Ω·cm, 500 μm thick, single-sided polish, test grade), muscovite mica with composition KAl_2_(Si_3_Al)­O_10_(OH)_2_ (SJ Trading, A1 special grade), as well as hydrophobized silica wafers by functionalization with silane (see SI.03 for the functionalization procedure). All the substrates except mica were cleaned by sonication in the ultrasonic bath in acetone (99%), ethanol (99%), and *Milli-Q* water (18.2 MΩ·cm at 25 °C) for 10 min each, then rinsed with *Milli-Q* water, and dried using a stream of nitrogen. Mica sheets were cleaved by insertion of a sharp needle tip into the edge of the sheet prior to the evaporation experiment. More detailed information on the substrates, their structures, and surface chemistry can be found in SI.04. The surface wetting properties such as contact angles and surface-free energies of glass and silicon substrates with ZnO droplets and standard solvents have been reported in ref 11

### GIXRD Experimental Setup

*In situ* synchrotron grazing incident X-ray diffraction (GIXRD) experiments were performed at Beamline I07 (Surface and Interface Diffraction) at Diamond Light Source, U.K.,^54^ where a monochromatic X-ray beam of energy *E* = 12.5 keV (λ = 0.9919 Å, size ∼ 100 × 300 μm^2^) and a Pilatus 2 M detector (Dectris) were used. The experimental setup is schematically shown in Figure 2. The sample stage was enclosed in a chamber. A fixed metal arm inside the chamber supported tubing, which was connected to a syringe pump, facilitated remote dispensing of a single droplet (Figure 2b). The substrate (∼1 × 1 cm^2^) was placed upon an aluminum block which was mounted on a hexapod for positioning (Figure 2c). In the adopted coordinate system, the *y*-axis was along the direction of the X-ray beam, with the detector surface and the sample surface in the *xz*- and *xy*-planes, respectively (Figure 2a). The *q* range of the setup was calibrated using silver behenate (AgC_22_H_43_O_2_), with a detector-to-sample distance of 0.396 m. The residual patterns on different substrates using different ZnO particles have been previously reported.^11^ The experimental focus here was to capture the rapid emergence of surface crystals as the droplet dried, leading to an ultimate surface pattern.

**Figure 2:**
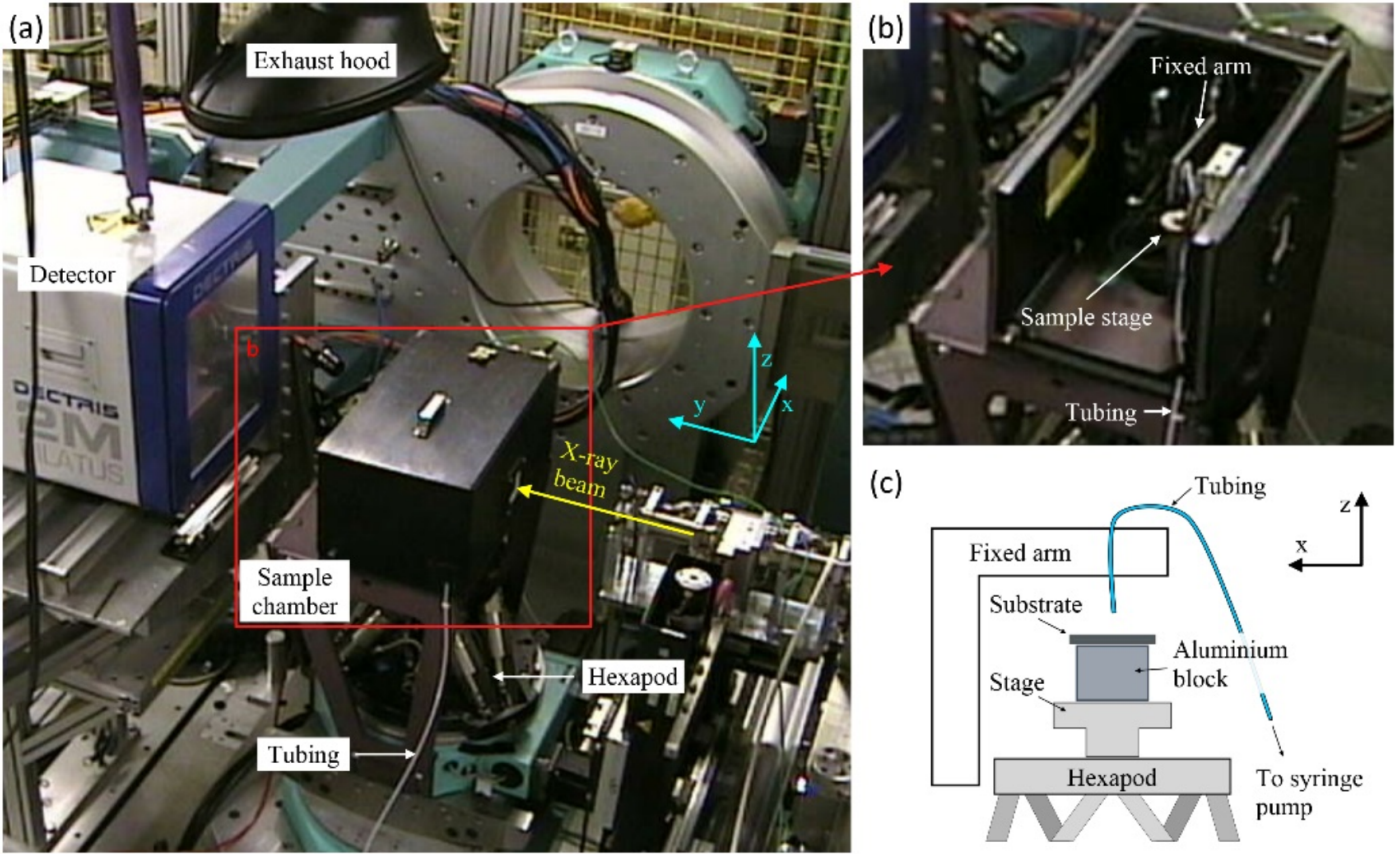
Experimental setup for *in situ* GIXRD at Beamline I07, Diamond Light Source, U.K.. (a) Sample chamber placement; and (b, c) the sample stage with the droplet dispensing mechanism, with the resistive heating facilitated *via* the aluminum block.

### Experimental Procedures

A typical experimental procedure consisted of initial alignment, deposition of the droplet, and a time-resolved GIXRD scan. The substrate (Figure 2c) was first aligned by a series of *height* (along the *z*-axis) and *theta* (θ, rotation around the *x*-axis) scans. Then, the substrate was initially tilted around the *x*-axis by 0.7° (the X-ray beam incident angle with the substrate). Subsequently, ∼60 μL of the nano/microfluid was dispensed from the tubing and deposited onto the substrate. Immediately after this, the *time scan* was performed with the diffraction data continuously collected at the time interval of 4–5 s as the drop was drying on the substrate, which typically lasted for *∼*200–300 s. The dried sample was then scanned laterally along the *x*-axis (referred to as *lateral scan*), which did not reveal any structural inhomogeneities. The procedure was undertaken under ambient relative humidity (RH ∼50%). The computing codes for the experimental scripts for these scans are listed in SI.05.

### Data Processing and Analysis

Raw two-dimensional (2D) diffraction patterns (manifesting as isotropic rings) were processed using *pygix*, a generic Python library designed for the reduction of grazing-incidence and fiber X-ray scattering data,^55^ which uses *pyFAI*, another Python library developed to perform One-dimensional (1D) azimuthal or 2D radial integrations of diffraction images.^56^ The recorded diffraction patterns were reduced to one-dimensional intensity *vs q* line profiles, where *q* = 4π sin­[(2θ)/2] /λ is the momentum transfer, λ is the X-ray radiation wavelength, and 2θ is the scattering angle. Baseline corrections of the diffraction line profiles were performed according to the penalized asymmetric least-squares algorithm.^57^ Selection of the algorithm parameters, comparison with the raw data, and the identification of the integration artifacts resulting from the integration are discussed in SI.06.

## Results and Discussion

### In-house ZnO Nanofluid

Figure 3 shows an example of the *in situ* GIXRD data from the drying of a droplet of the *in-house ZnO nanofluid* on a *glass* substrate. This contrasts with the control GIXRD data from the bare substrates only (without the nanofluid; Figure S6). These diffractograms are produced by projecting real detector images onto the reciprocal space coordinates *q*_*z*_ (out-of-plane or perpendicular) and *q*_*xy*_ (in-plane or parallel). At the initial stage of evaporation, an intense diffuse peak arising from the short-range order interactions in the bulk liquid^58^ was observed at *q* ≈ 12.7 ± 1.0 nm^–1^. This liquid peak was visible in all the measurements and its intensity diminished as the evaporation progressed (*cf*. the relative intensities in the color bars in Figure 3a, b) and eventually disappeared when evaporation was close to completion at *t* ∼50 s. In addition, the diffraction peaks at positions *q* = 4.47, 6.99, 7.77, and 8.90 nm^–1^ emerged as soon as evaporation began, becoming clearly visible in Figure 3b. The broad diffuse scattering ring between 14 and 24 nm^–1^ in Figure 3c was from the glass substrate (*cf*. Figure S6a,b).

**Figure 3:**
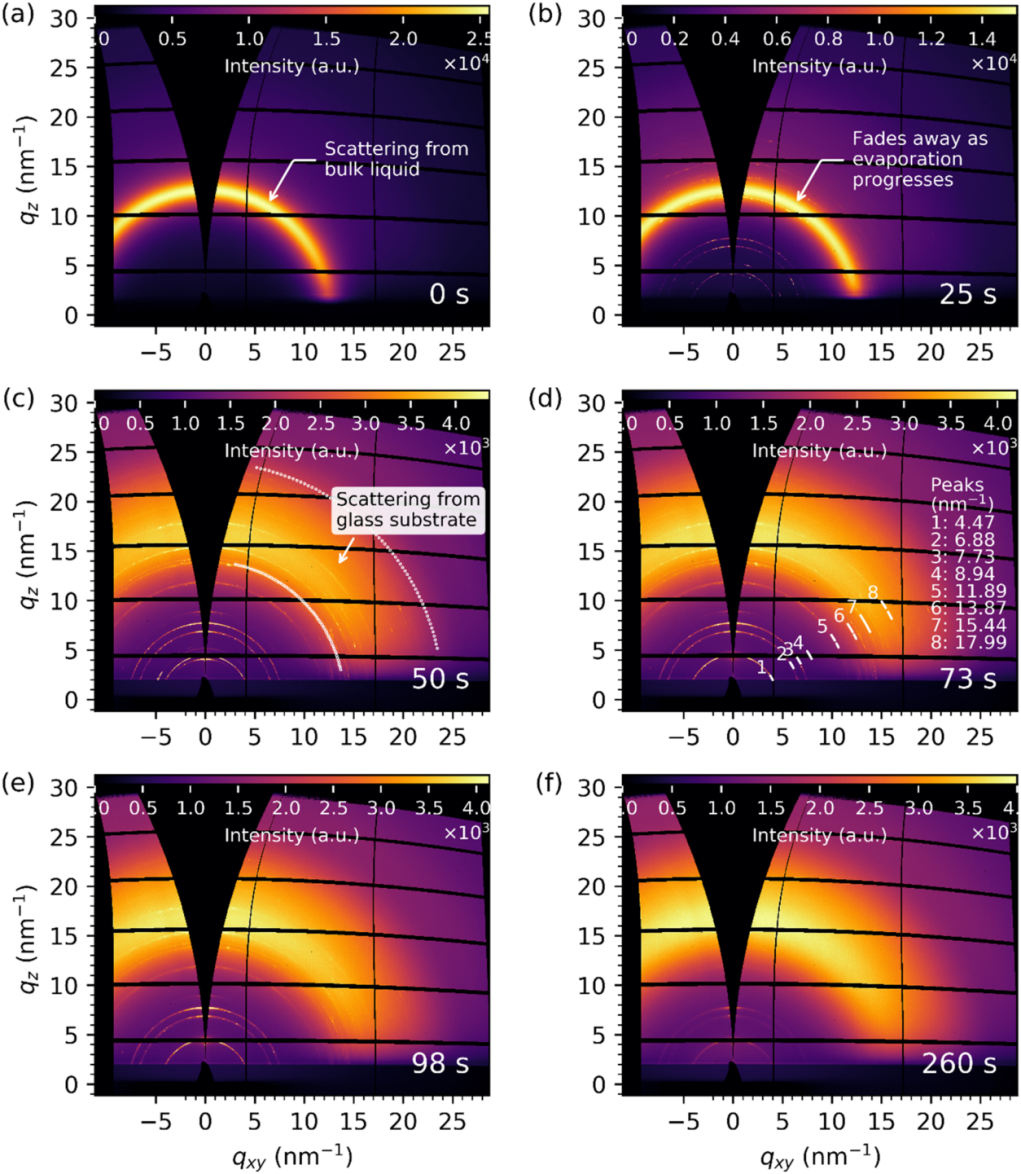
Time-resolved diffractograms from *in situ g*razing incident X-ray diffraction (GIXRD) data from an evaporating droplet containing the in-house ZnO nanofluid on a glass substrate. (a–f) Detector images taken at different evaporation times (0–260 s, as labeled) projected onto reciprocal space coordinates: *q*_*z*_ (out-of-plane) and *q*_*xy*_ (in-plane). Intensity scale color bars are placed at the top of each diffractogram, where au stands for arbitrary units. The scattering from the bulk liquid was visible in the first 25 s (a, b). The diffraction rings due to the surface zinc hydroxide crystals had already appeared at 25 s. Diffuse scattering from the glass substrate is outlined in (c). (d) Calculated peak positions for the surface structures inside of the drying droplet, and the diminishing peak intensities in (e–f) were likely caused by the X-ray beam sample damage.

Distinct diffraction rings are numbered with their positions in Figure 3d. The peaks correspond to interplanar spacing (*d* = 2π/*q*) of 1.41, 0.91, 0.81, 0.70, 0.53, 0.45, 0.41, and 0.35 nm, respectively. The three peaks labeled as 1 (4.47 nm^–1^), 2 (6.88 nm^–1^), and 4 (8.94 nm^–1^) can be indexed as (002), (003), and (004) arising from the stacked zinc hydroxide layers with interlayer anionic species with the interplanar *d*-spacing of 2.79 ± 0.02 nm as expected.^10,59,60^ The diffraction ring from (001) would appear at *q* ≈ 2.23 nm^–1^, and this *q* range was partially obscured by the beam stop. Peaks 3 (7.73 nm^–1^) and 7 (15.44 nm^–1^) in Figure 3d are also equally spaced and may result from other stacked crystallographic planes. However, their precise crystallographic origin remains to be determined. Figure 3e–f shows the zinc hydroxide diffraction patterns persisting at later stages of the drying process with time elapsed *t* = 98 and 260 s, respectively. The intensity of the diffraction rings, excluding the diffuse scattering from the glass substrate, diminished over time likely due to beam damage from the prolonged X-ray exposure.

Such time-resolved GIXRD data allow insights into the structural evolution of the surface pattern upon the rapid evaporation process. The diffraction patterns (Figure 3) were integrated over the azimuthal angle to generate the streak plot in Figure 4a, which shows integrated diffraction intensity *vs q* over time *t*. As soon as a drop of the ZnO nanofluid was cast on the substrate, diffraction peaks labeled as 1 (4.47 nm^–1^), 2 (6.88 nm^–1^), 3 (7.73 nm^–1^), 7 (15.44 nm^–1^), and 8 (17.99 nm^–1^) emerged, indicating the rapid formation of the layered zinc hydroxide (LZH) structures within *t ≈* 0–10 s. Selected diffraction line profiles, marked by horizontal dashed lines in Figure 4a, related to different evaporation times of 6, 25, and 98 s are plotted in Figure 4b (and enlarged views in Figure 4c,d). Sharp and intense LZH peaks 1–3 and less intense peaks 4 and 7–9 were visible as early as at *t* = 6 s. Other LZH peaks 4–6 became more intense after 10–15 s (Figure 4c). The peaks labeled with numbers 8 (17.99 nm^–1^) and 9 (19.87 nm^–1^) retained low intensity through the entire drying process up to 260 s in contrast to peaks 1–3, and to some extend 4–7. They were likely the higher order reflections from the (00*l*) planes, i.e., (008) and (009) for peaks 8 and 9, respectively.^60,61^

**Figure 4:**
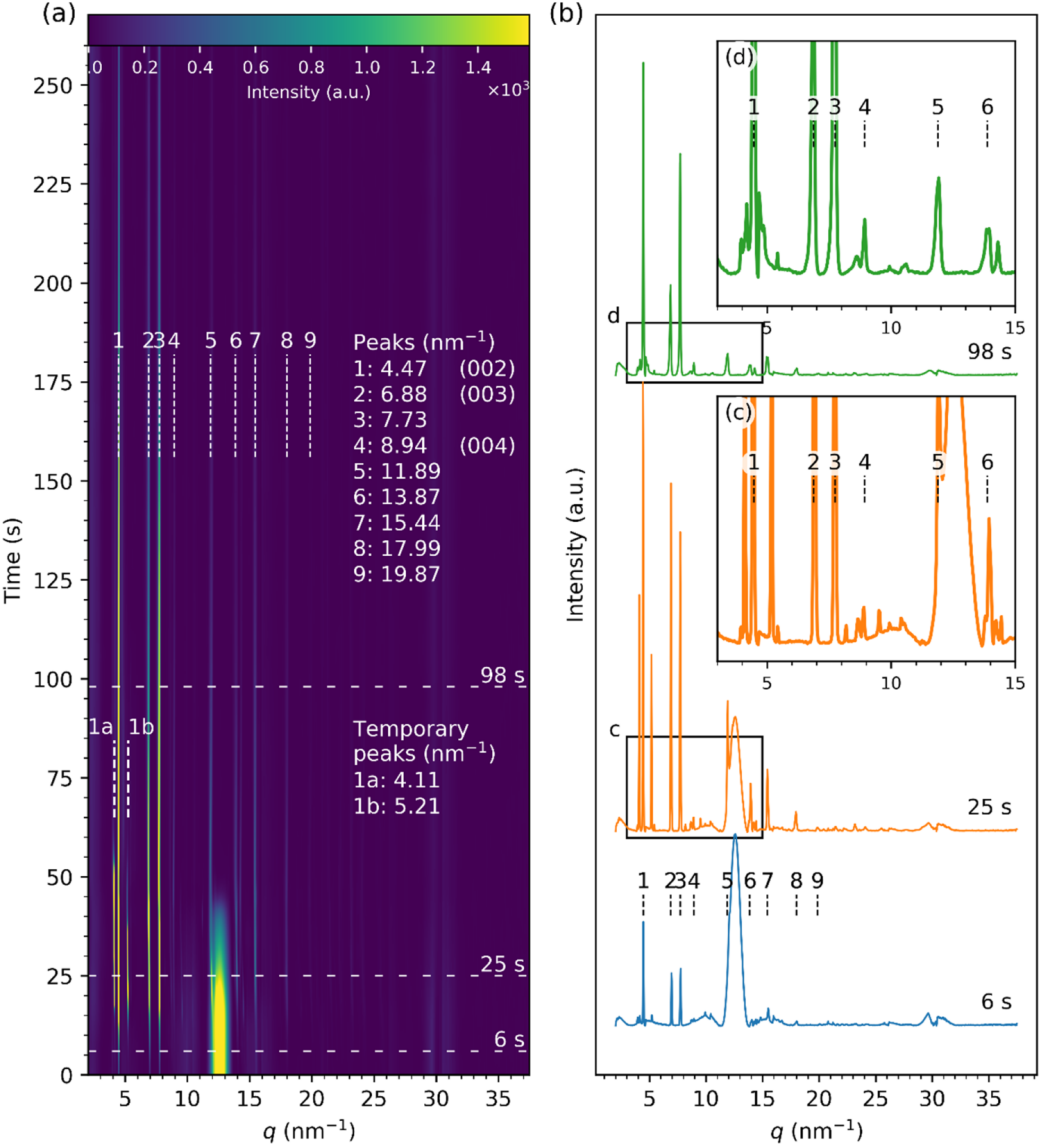
X-ray diffraction data from the evaporating droplet of the in-house ZnO nanofluid on a glass substrate produced by azimuthal integration of the diffractograms are presented in Figure 3. (a) A streak plot shows how the intensity of the scattering signal changes with the evaporation time. Dashed horizontal lines mark the positions of the line profiles in (b). Vertical lines numbered 1 – 9 show the peak positions fitted at *t* = 73 s and the lines labeled 1a and 1b at *t* = 25 s, also listed in (a). (b) Selected line profiles with enlarged sections in insets (c, d) show the data at *t* = 6, 25, and 98 s in detail. Note that the relative intensities between the sharp LZH and the bulk liquid peaks are different from Figure 3 as the integrated profiles in Figure 4 have been baseline corrected using the penalized asymmetric least-squares algorithm^57^ for better contrast and scattering signal visualization. The correction is discussed in detail in SI.06.

There are no peaks for the zinc oxide crystals. These would be expected at *q* values of 22.39, 24.21, 25.45, and 32.94 nm^–1^ for (100), (002), (201), and (102) planes, respectively (Powder Diffraction File (PDF) number assigned by the International Centre for Diffraction Data: 01-075-0576). The absence of any ZnO peaks also indicates no deposition of the dispersed ZnO nanoparticles at the interface. Interestingly, two additional diffraction peaks labeled as 1a and 1b (“temporary” peaks) located at *q* = 4.11 and 5.21 nm^–1^, respectively, became very intense at *t* ∼ 15–60 s and then faded as the evaporation progressed. These are tentatively attributed to nanocrystals comprising layered structures intercalated with solvent and isobutylamine. Such transient nanocomplexes preceded LZH peak emergence and thus could be considered as precursor to LZH formation or represent intermediate complexes between ZnO and LZH. The intense scattering signal around *q* ≈ 12.7 ± 1.0 nm^–1^ arising from the bulk liquid^58^ diminished after *t ≈* 45–50 s, corresponding to near-complete evaporation.

Figure 5 shows the time-resolved diffraction data of the *in-house ZnO nanofluid* drop evaporating on *an unmodified silicon (Si)* substrate. As previously observed, no ZnO diffraction peaks were observed, suggesting no ZnO nanoparticle deposition on the surface throughout the evaporation process. Intriguingly, the first indication of the structural changes in the evaporating droplet are “temporary” peaks labeled as 1a (4.15 nm^–1^) and 1b (5.21 nm^–1^) emerging as early as *t* = 14 s and persisting until *t* ∼ 160 s (Figure 5b,d). After a further 6 s, the rest of the peaks attributed to the layered zinc hydroxide structures labeled as 1–9 appeared; however, their intensities were much smaller than peaks 1a and 1b but grew gradually with time (Figure 5b,c). At *t* ∼140 s, most of the liquid had evaporated, marked by the disappearance of the bulk liquid peak at *q* = 12.7 ± 1.0 nm^–1^. At that time, the intensity of peak 1 (4.50 nm^–1^) and indexed as (002) of zinc hydroxide became significantly stronger than that of the 1a and 1b peaks. The average interplanar *d*-spacing calculated from the position of the 1, 2, and 4 peaks was 2.77 ± 0.02 nm.

**Figure 5:**
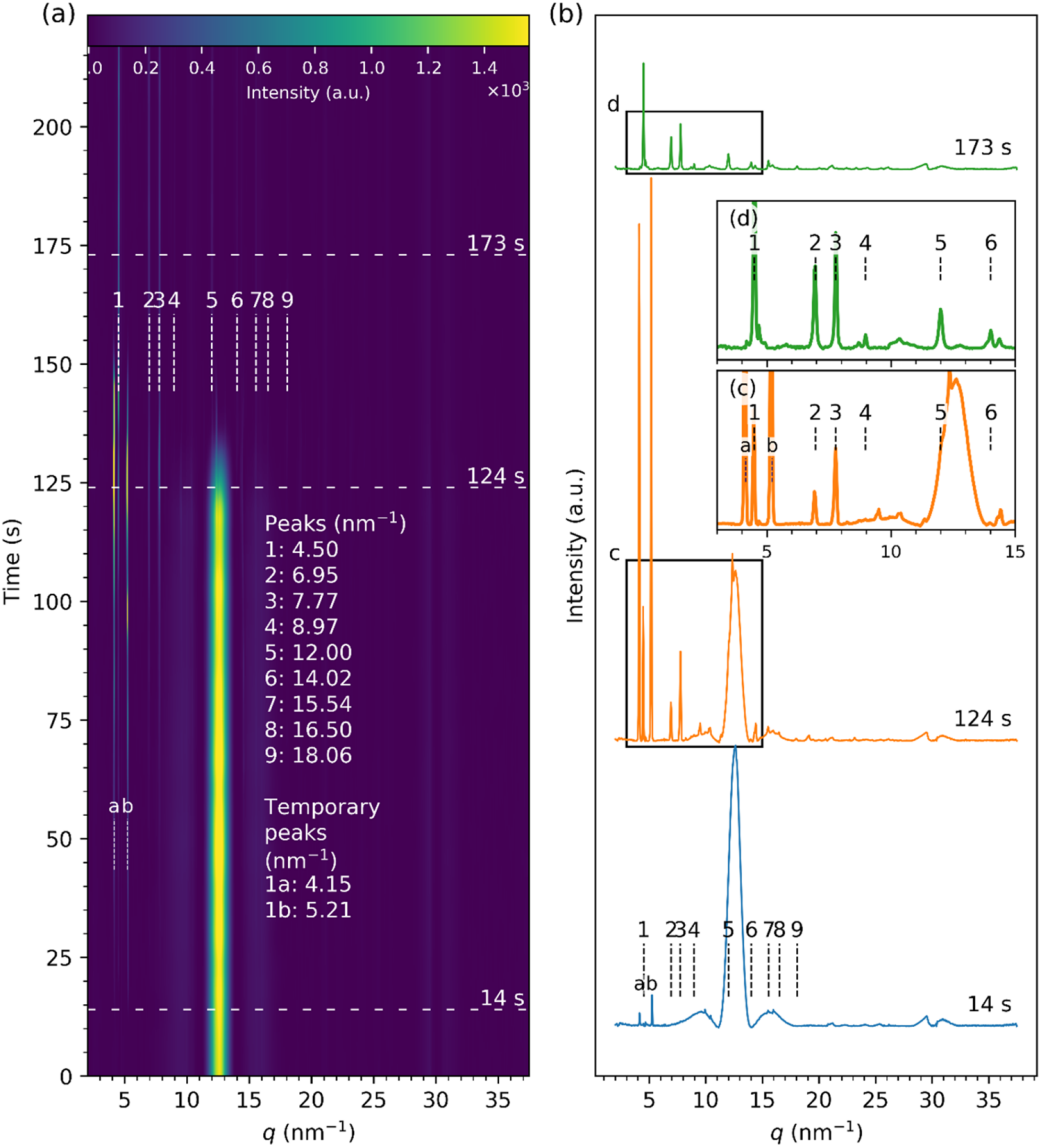
X-ray diffraction data from the evaporating droplet of the *in-house ZnO nanofluid* on an *unmodified silicon* substrate: (a) Streak plot shows how the intensity of the scattering signal changes with the evaporation time. Dashed horizontal lines mark positions of line profiles that are shown in (b). Vertical dashed lines labeled with numbers 1 – 9 show the positions of peaks fitted to the data; (c, d) insets show lower *q* range of the diffraction profiles at *t* = 124 and 173 s.

The time-resolved diffraction data for a drying droplet of the *in-house ZnO nanofluid* on a *silanized (hydrophobic) Si* substrate are shown in Figure 6. Diffraction peaks 1 – 3 attributed to the LZH structures appeared within 4 s of evaporation. Other LZH peaks (4–7 and 9) emerged 20–30 s after and became more intense with time. Similarly to the evaporation from the two other substrates, glass (Figures 3 and[Fig fig4]) and unmodified silicon (Figure 5), additional peaks close to the first LZH (4.47 nm^–1^) peak, labeled as 1a (4.12 nm^–1^) and 1b (5.20 nm^–1^), appeared at *t* ∼ 50–60 s and grew more intense with time, then disappeared at *t* ∼ 174 s, just after the completion of evaporation marked by the disappearance of the bulk liquid peak (*q* = 12.7 ± 1.0 nm^–1^). The intensity of the first LZH peak at 4.47 nm^–1^ was much higher than that of 1a and 1b, similar to the observations on glass (Figure 4) but in contrast to the unmodified Si substrate (Figure 5), possibly due to higher adsorption of LZH complexes on the surface.

**Figure 6:**
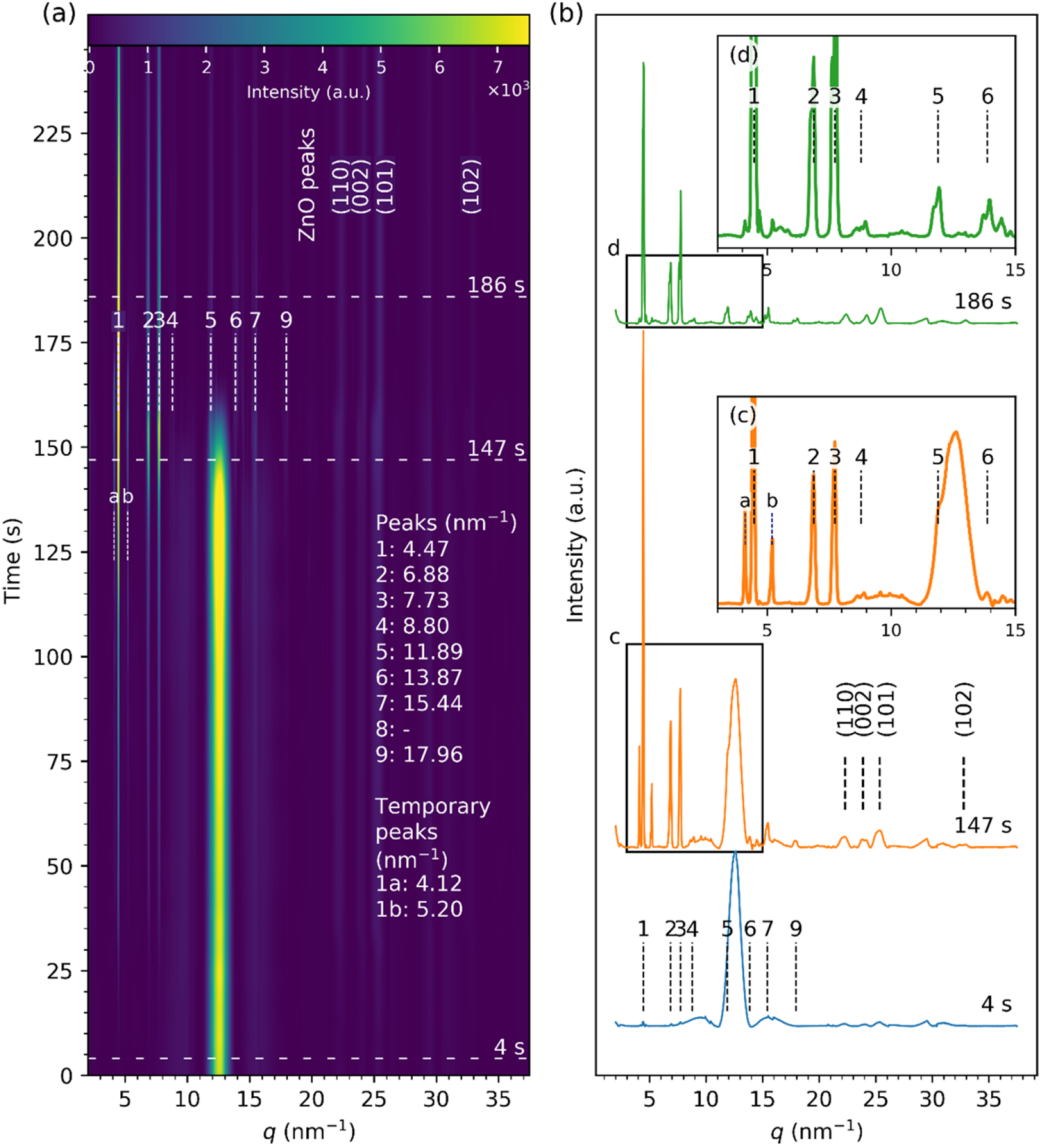
GIXRD data from the evaporating droplet of the *in-house ZnO nanofluid* on a *silanized Si* substrate: (a) Streak plot shows how the scattering intensity changes as a function of the evaporation time *t*. Vertical dashed lines labeled with numbers 1–7 and 9 show the fitted positions, and the horizontal dashed lines mark the times for the selected profiles shown in (b). (c, d) Enlarged view of the lower *q* range of the diffraction profiles at *t* = 147 and 186 s.

The peaks labeled as 1 (4.47 nm^–1^), 2 (6.89 nm^–1^), and 4 (8.80 nm^–1^) resulted from the stacked (00*l*) planes of the layered zinc hydroxide structures give an average interplanar *d*-spacing of 2.80 ± 0.03 nm. In contrast to the observations above (Figures 3–5), ZnO diffraction peaks at 22.29, 23.89, 25.34, and 32.76 nm^–1^, indexed as (110), (002), (101), and (102), respectively, were present throughout the droplet drying. However, their intensity was relatively low when compared with the main LZH peaks at the low *q* range. The peak broadening was observed for all ZnO peaks, with their coherence lengths (see SI.09) of 8.9 ± 3.5 nm for (101), 10.8 ± 6.0 nm for (002), 9.6 ± 2.3 nm for (101), and 9.6 ± 0.2 nm for (102), and the average *L*_a_ of 9.7 ± 2.6 nm, smaller than the 12.6 ± 2.6 nm for the in-house ZnO nanoparticles. This indicates that some unreacted ZnO nanoparticles deposited on the hydrophobic surface, where they were retained, throughout the evaporation process, in contrast to the hydrophilic substrates. This could be understood in terms of the role of trace amounts of water in the ZnO dissolution,^7,8^ with a more hydrophobic surface less susceptible to mediating hydrolysis in the process. The diffraction peaks of Zn­(OH)_2_ emerge as soon as the drop was cast on the hydrophilic substrate, while on the hydrophobic substrate, the diffraction peaks from ZnO were more visible at this stage. The ZnO nanoparticle dissolution process would require moisture, and in this case, trace amounts of water on the surface. It is conceivable that the hydrophilic surface facilitates this process more readily, compared to the hydrophobic surface.

### Droplet of ZnO Nanopowder Dispersion

GIXRD data were also collected for the ZnO nano/microfluid prepared from the commercially sourced ZnO nanopowder (68.3% of crystals between 36 and 142 nm; *cf*. SI.02). Compared to the in-house-synthesized ZnO nanoparticles above, the nanopowder had a higher crystallinity with a larger *L*_a_ ∼ 60 ± 11 nm. Our pervious SEM images showed the residual ZnO particles at the end of the evaporation process due to incomplete dissolution of such ZnO nanopowders.^10^ Figure 7 presents results from ZnO nanofluid drying on an unmodified Si substrate. In contrast to the in-house ZnO nanofluid, the strong ZnO diffraction peaks produced by the wurtzite structure of larger crystals appear at the start of the evaporation at *t* = 0 s. These ZnO peaks were located at *q* = 22.43, 24.24, 25.48, and 32.97 nm^–1^, indexed as (110), (002), (101), and (102), respectively, and persisted throughout the drying process. After *t* = 44 s, the intensity of the ZnO peaks increased, coinciding with the diminishment of the liquid scattering ring (*cf*. Figure 7a), which could be attributed to the onset of the droplet thinning. In addition, the peaks due to the LZH structures numbered 1 at *q* = 4.50 nm^–1^ and 2 at *q* = 6.99 nm^–1^ started emerging at *t* = 30 s. This contrasts with the much faster emergence in the case of the in-house ZnO nanofluid (Figures 3–6), which can be related to slower dissolution of the commercially acquired ZnO nanopowder due to their size, morphology, and crystallinity.^10^

**Figure 7:**
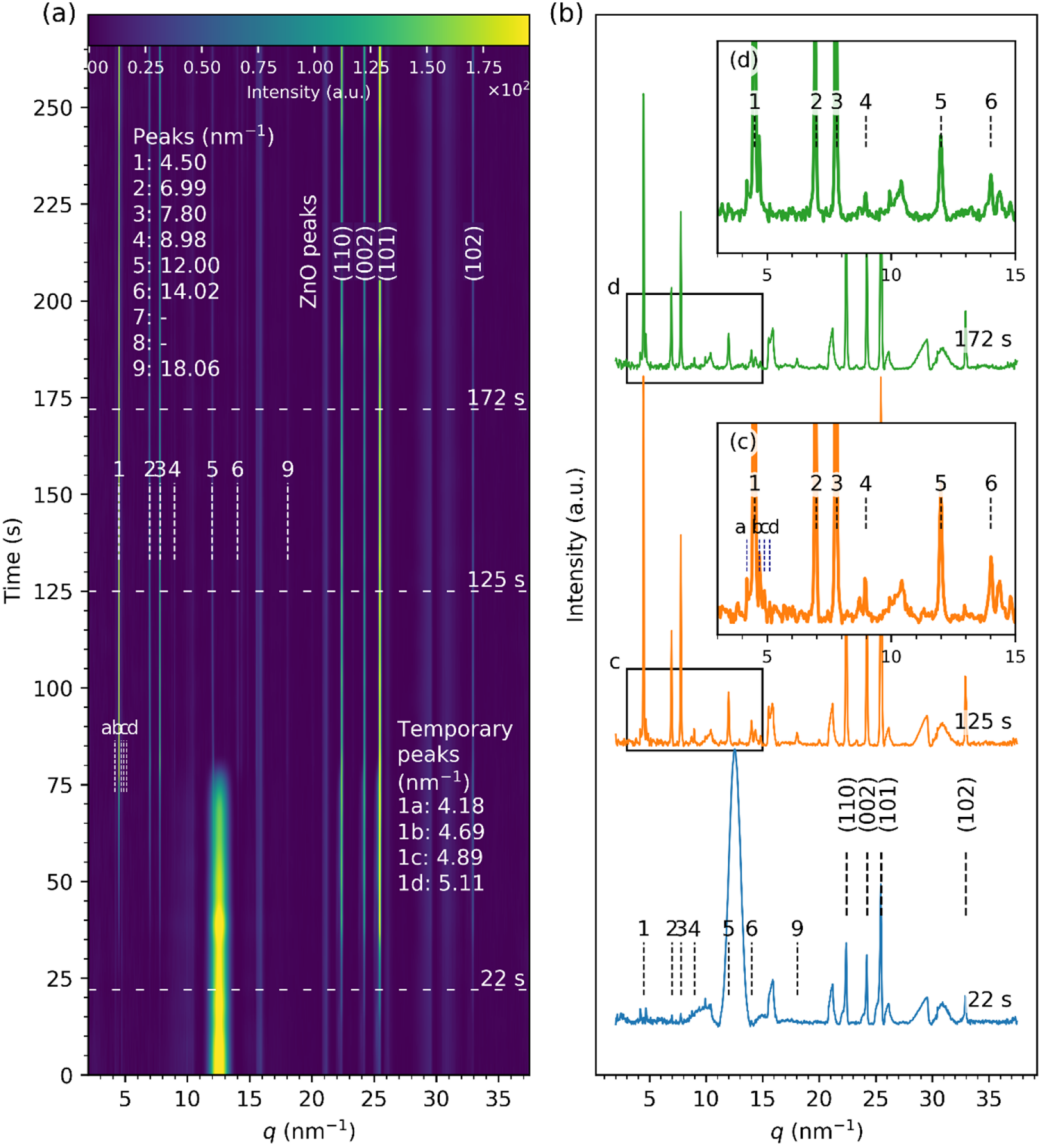
Time-resolved GIXRD data from a droplet of ZnO nanopowder dispersion drying on an unmodified Si substrate: (a) Streak plot of diffraction line profiles with peaks annotated. (b) Snapshots of selected diffraction profiles at different times, *t* = 22, 25, and 172 s of the evaporation process. (c, d) Enlarged views of the data in the lower *q* range.

The average coherence length of ZnO peaks reduced from ∼60 to ∼43 nm (cf. SI.09). Dissolution of ZnO nano/microparticles has been discussed in ref 10. It was shown that the morphology of the ultimate residual pattern from the evaporation of ZnO nanofluids could be controlled by varying the crystallinity, shape, and size of the starting ZnO nanoparticles, which affected the nanoparticle dissolution process during evaporation, the key step in the evaporation-induced self-assembly process.

The intensity of the LZH peaks increased and became comparable with the ZnO peaks at *t* ∼ 44 s, suggesting the abundance of both undissolved ZnO nano/microcrystals and rapidly forming LZH structures on the surface. The bulk liquid peak at *q* = 12.7 ± 1.0 nm^–1^ disappeared after ∼80 s (most of the solvent was lost due to evaporation), at which the rest of the LZH peaks labeled as 3 (7.80 nm^–1^), 4 (8.98 nm^–1^), 5 (12.00 nm^–1^), 6 (14.02 nm^–1^), and 9 (18.06 nm^–1^) also became clearly visible. The average interplanar *d*-spacing calculated from the positions of peaks 1, 2, and 4 was 2.76 ± 0.03 nm. In contrast to the in-house ZnO nanofluid dried on glass (Figure 4), there were no distinguishable peaks 7 and 8. Interestingly, the peaks around the (002) LZH peak labeled as 1, termed as “temporary” for the in-house synthesized ZnO nanofluid (Figures 3,4), appeared at 4.18, 4.69, 4.89, and 5.11 nm^–1^ (labeled as 1a-d, respectively) as early as *t* = 0 s. These peaks retained constant shapes and intensities throughout, with intensities negligible compared to the first LZH peak at 4.50 nm^–1^. They are similarly attributed to intermediate molecular/nanocrystal complexes resulting from rapid ZnO dissolution, preceding the transformation into Zn­(OH)_2_ nanocrystals.

Figures S14–S16 (in SI.07) show diffractograms collected for the ZnO nanopowder nano/microfluid evaporated on other substrates: glass, silanized Si, and muscovite mica, respectively. The diffraction peaks at *q* = 22.38, 24.21, and 25.45 nm^–1^ appeared in every diffractogram from the start of the evaporation (*t* = 0 s). These are related to (100), (002), and (101) of the crystallographic planes of ZnO (PDF 01-075-0576) and resulted from the undissolved ZnO nano/microparticles deposited on the surface inside the drying droplet, similarly for the ZnO powder nano/microfluid (Figures S17–S20 in SI.07). In all cases, the low *q* range peaks could be observed, indicating the formation of LZH structures, similar to that shown in Figure 7.

### Evaporation of Droplet of ZnO Powder Dispersion

Evaporation of droplets containing ZnO powder (68.3% of crystals between 61 and 291 nm, see SI.02) dispersion was also studied. These particles also had a high crystallinity, evident from their large coherence length value of ∼57 ± 8 nm (reduced to ∼51 ± 7 during evaporation), leading to slower dissolution and thus coexistence of ZnO and LZH structures throughout the evaporation process, which is qualitatively very similar to the results from the ZnO nanopowder above. These results are shown in Figures S17, S20 in SI.07.

## Summary and Conclusions

In this study, we have applied *in situ* grazing-incidence X-ray diffraction to probe the buried interface between a reactive ZnO nanofluid sessile drop and the substrate as the droplet underwent rapid evaporation. While the final residual surface patterns and the pattern formation mechanisms have been previously reported, the high temporal resolution of synchrotron X-rays here allowed us to ascertain of the emergence of Zn­(OH)_2_ surface crystals from the onset of the evaporation and their rapid evolution into the final residual surface pattern. We compared in-house synthesized ZnO nanoparticles, with commercially sourced ZnO nanopowder and ZnO powder with higher crystallinity, as well as different substrates (i.e., glass slides, mica, silica, and silanized-silica).

Using the *in situ* grazing incident X-ray diffraction measurements, we were able to track the structural changes occurring in the drying reactive nano/microfluid droplets, leading to the formation of hierarchical surface structures. The rapid formation of the layered zinc hydroxide LZH structures characterized by the average interplanar *d*-spacing of 2.78 ± 0.02 nm was confirmed. This occurred almost immediately as the evaporation was initiated for the ZnO nano/microfluids prepared from the in-house-synthesized ZnO nanoparticles; for the commercially sourced ZnO nanopowder and ZnO powder with higher crystallinity and thus slower dissolution, the LZH structures emerged at a later stage, i.e., after some 30 s. In addition, a formation of an intermediate layered phase was evident from the transient peaks that appeared only during the initial stage of the evaporation process and then diminished as the LZH structures formed. The identity and structure of the “intermediate layered phase” should be a subject of future investigation. The average interlayer *d*-spacing calculated for LZH was 2.80 ± 0.02 nm. The surface chemistry of the substrate did not alter the mechanistic process of the ZnO to LZH transformation; instead, the more hydrophobic silanized Si substrate appeared to affect the kinetics of ZnO dissolution. However, we have reported the influence of the substrate on the final morphology of as-formed residual patterns and also on induced Bénard–Marangoni cells inside the drying droplet before.^11^ Hence, while the substrate surface chemistry does not affect the chemical transformation of reactive zinc oxide particles into zinc hydroxide fibrous network,^8^ it affects the solvent flow and thus the ultimate microscopic morphology in the residual pattern.^11^ These mechanistic insights revealed by the *in situ* grazing incident X-ray diffraction studies will facilitate further discussion relevant to the novel evaporation-induced self-assembly process, involving reactive nanofluids and other applications of reactive nanofluids, e.g., as coolants. Future experiments using synchrotron GIXRD could further probe the effects of relative humidity, different solvents, and other types of reactive nanofluids. It will also be useful to explore combining GIXRD with other *in situ* multiscale, time-resolved characterization techniques which would further enhance the mechanistic understanding of the reactive nanofluid evaporation and associated pattern formation.

## Supplementary Material



## Notes

Additional details on pattern formation mechanism; experiment materials; programming scripts for data collection; additional XRD diffractograms; and process for XRD profile baseline correction, and coherence length calculations (PDF)

## #

Purdy & Figg Ltd, Blackwell Hall, Blackwell Hall Lane, Chesham, HP5 1TN, U.K

## ∇

Johnson Matthey, Blount’s Court, Sonning Common, Reading RG4 9NH, U.K..

## ○

European Spallation Source, The ESS Campus, Lund, Sweden, SE-221 00; ISIS Neutron and Muon Source, Science and Technology Facilities Council, Rutherford Appleton Laboratory, Harwell, Oxford, Didcot, OX11 0QX, U.K.; Department of Chemistry, University of Bath, Claverton Down, Bath, Avon BA2 7AY, U.K..
