# Supplementary material for: Evaporation of a Reactive Nanofluid Sessile Drop: Capturing Rapid Emergence of Surface Crystals with In Situ Synchrotron X‑ray Diffraction
Source: Langmuir. 2025 Jul 9;41(28):18730–40. doi: 10.1021/acs.langmuir.5c01989 (PMC12288063; doi:10.1021/acs.langmuir.5c01989)
Supplement: Supplementary file 1 [file la5c01989_si_001.pdf]

Supplementary Information

Evaporation of a reactive nanofluid sessile drop:

Capturing rapid emergence of surface crystals with in-situ synchrotron X-ray diffraction

Patryk Wąsik[a,*] *Anna Slastanova*[b,&], *Jacek M. Wąsik*[c,d], *Tim Snow*[b,e], *Alexander Gerrit de Bruin*[b,⊥], *Thomas Arnold*[e,#], *and Wuge H. Briscoe*[b,*]

[a] National Synchrotron Light Source II, Brookhaven National Laboratory, Upton, New York 11973, USA

[b] School of Chemistry, University of Bristol, Cantock's Close, Bristol, BS8 1TS, UK

[c] Bristol Centre for Functional Nanomaterials, HH Wills Physics Laboratory, University of Bristol, Tyndall Avenue, Bristol, BS8 1TL, UK

[d] School of Physics, HH Wills Physics Laboratory, Tyndall Avenue, Bristol, BS8 1TL, UK

[e] Diamond Light Source, Diamond House, Harwell Science and Innovation Campus, Didcot, Oxfordshire, OX11 0DE, UK

[&] Present address: Purdy & Figg Ltd, Blackwell Hall, Blackwell Hall Lane, Chesham, HP5 1TN, UK

[⊥] Present address: Johnson Matthey, Blount's Court, Sonning Common, Reading RG4 9NH, UK

[#] Present address: European Spallation Source, The ESS Campus, Lund, Sweden, SE-221 00; ISIS Neutron and Muon Source, Science and Technology Facilities Council, Rutherford Appleton Laboratory, Harwell, Oxford, Didcot, OX11 0QX, UK; Department of Chemistry, University of Bath, Claverton Down, Bath, Avon BA2 7AY, UK

[*] Corresponding authors: wuge.briscoe@bristol.ac.uk (WHB) and pwasik@bnl.gov (PW)

Table of Contents

SI.01: Mechanism 3

Revised mechanism for Bénard-Marangoni (BM) dendrite formation 4

Cellular patters with dendritic morphologies 6

Further evaporation and crystallisation 8

SI.02: Particles 8

SI.03: Substrates 11

Silanation of Si wafer procedure 11

ZnO nanorods on glass synthesis 13

GIXRD control scans of substrates 13

SI.04: Information about substrates 15

Microscope Glass Slides 15

Silicon Wafer Substrates 17

Mica Substrates 19

SI.05: Scripts 20

SI.06: Baseline correction of diffraction profiles 22

SI.07: Time resolved grazing incidence diffractograms 29

Droplet of ZnO nanopowder dispersion 29

Droplet of ZnO powder dispersion 31

SI.08: Coherence length calculations 36

SI.09: References 38

SI.01: Mechanism

The first attempt to explain the mechanism behind the formation of hierarchical residual surface patterns from evaporation of a reactive ZnO nanofluid sessile drop was made by Wu *et al.* in 2014 [1]. When a droplet containing ZnO nanorods dispersed in a mixture of isobutylamine and cyclohexane is placed on a substrate, rapid in-take of water molecules from air onto the surface of isobutylamine-coated ZnO nanorods would initiate reaction S1:

$$\text{i-}C_4H_{11}N + H_2O \leftrightarrows \text{i-}C_4H_{12}N^+ + OH^-. \qquad \text{(S1)}$$

The hydration-dissolution of ZnO nanorods proceeds afterwards, likely starting from the (0001) facets located at the tips of the nanorods due to their exposed OH^- groups:

$$ZnO_{(s)} + \text{i-}C_4H_{12}N^+ + OH^- + H_2O \leftrightarrows Zn(OH)_3^- \cdot {}^+N\ \text{i-}C_4H_{12(aq)}. \qquad \text{(S2)}$$

Convective flows shuttle liquid $Zn(OH)_3^- \cdot {}^+N$ i-$C_4H_{12(aq)}$ away from the nanorods, allowing their further dissolution. As the evaporation progresses, the solution reaches its saturation point at the liquid-air interference towards the edge of the droplet, where crystallisation of $Zn(OH)_2$ is initiated according to the reaction

$$Zn(OH)_3^- \cdot {}^+N\ \text{i-}C_4H_{12(aq)} \leftrightarrows Zn(OH)_{2(s)} + \text{i-}C_4H_{11}N + H_2O. \qquad \text{(S3)}$$

It is suggested that the driving force leading to the assembly of $Zn(OH)_2$ nanocrystals into long fibres was a combination of the solvent evaporation, crystallisation-mediated fingering instabilities at dewetting front of the drying droplet, and hydrogen bonding between nanocrystals. As isobutylamine is surface active and evaporates rapidly, the solutes, which include undissolved ZnO nanorods and liquid $Zn(OH)_3^- \cdot {}^+N$ i-C_4H_{12}, tend to localise and concentrate close to the liquid-air interface.

Crystallisation of $Zn(OH)_2$ is thus initiated at the vicinity of the liquid-air interface due to the increased solute concentration. The three-phase contact line between the solvent, wet $Zn(OH)_2$ nanocrystals, and air subsequently arises and recedes in the direction of fibre growth as evaporation dewetting proceeds. Surface tension gradient induced by fast solvent evaporation and concentration gradient in the proximity of nonequilibrium crystallisation growth provoke fingering instabilities at the receding dewetting front. The

instabilities originate from numerous crystallisation sites at the droplet surface and its perimeter propagating inwards.

The freshly formed $Zn(OH)_2$ nanoplatelets covered with isobutylamine and water interplay with each other and bond through hydrogen bonding. $Zn(OH)_3^{-}\cdot{}^{+}N$ i-C_4H_{12} in its supersaturated isobutylamine solution is transported towards and into the growth front of the fingering instabilities, congesting $Zn(OH)_2$ nanoplatelets at the finger front *via* sustained crystallisation and promoting the growth fingers into fibrous networks. This process would propagate from the liquid-air interface towards the substrate surface and from the perimeter to the centre of the droplet, giving rise to the fibrous hierarchical structure.

Revised mechanism for Bénard-Marangoni (BM) dendrite formation

A revised mechanism for the hierarchal residual patterns formation from evaporation of reactive ZnO nanofluid sessile drops was proposed in 2018 [2]. The suggested mechanism is expanded to account for the cellular patters with dendritic morphologies, formed within the central region of the residue that is surrounded by a peripheral coffee-ring band. The considerations of the structural hierarchy on nano-/micro-/macroscopic levels are based on time-resolved transmission electron microscopy (TEM) and cryo-TEM observations of the constituent nanostructures (with ~2 min temporal resolution) inside the droplet at different stages of the evaporation process and video microscopy studies of the capillary waves at the droplet surface. The authors used droplets containing 1 mg/mL ZnO nanoparticles (~5-10 nm) dispersed in a mixture of chloroform/methanol/isobutylamine (at ratios of 3.85:1.15:1).

At the initial stage of evaporation, the outward flow transports ZnO nanoparticles to the edge of the droplet where they undergo moisture assisted dissolution into amphiphilic isobutylamine-ZnOH complexes (iZMCs) with a core size *ca* 0.24 nm (**Figure S1**a-c), which then form solvated aggregates around 100 nm in size (**Figure S1**d and f). At the same time, the iZMC aggregates are being distributed along the drop surface by the Marangoni flow (**Figure S1**f). In this rapid process, most ZnO nanoparticles are dissolved and transformed into iZMC aggregates within a few minutes of evaporation (**Figure S1**a, b, d, and e). As the process progresses, iZMCs assemble into primary clusters with 2 – 5 nm in size within the initially formed aggregates (**Figure S1**e and i). The primary clusters are suggested to be micelle- or vesicle-like,

consisting of a polar core of isobutylamine, methanol, and water shielded from the adjacent nonpolar solvent. However, the authors emphasised that the exact composition and structure of the primary clusters was not known and would require further investigation. These primary clusters further assemble into a network of secondary clusters with ~30 nm in size (**Figure S1**j-l). The coalescence of secondary clusters leads to the formation of linear or branched aggregates, shown in red frames in (**Figure S1**j). It is suggested that the self-assembly of clusters is prominent at the droplet surface due to their surface activity and is also driven by evaporation.

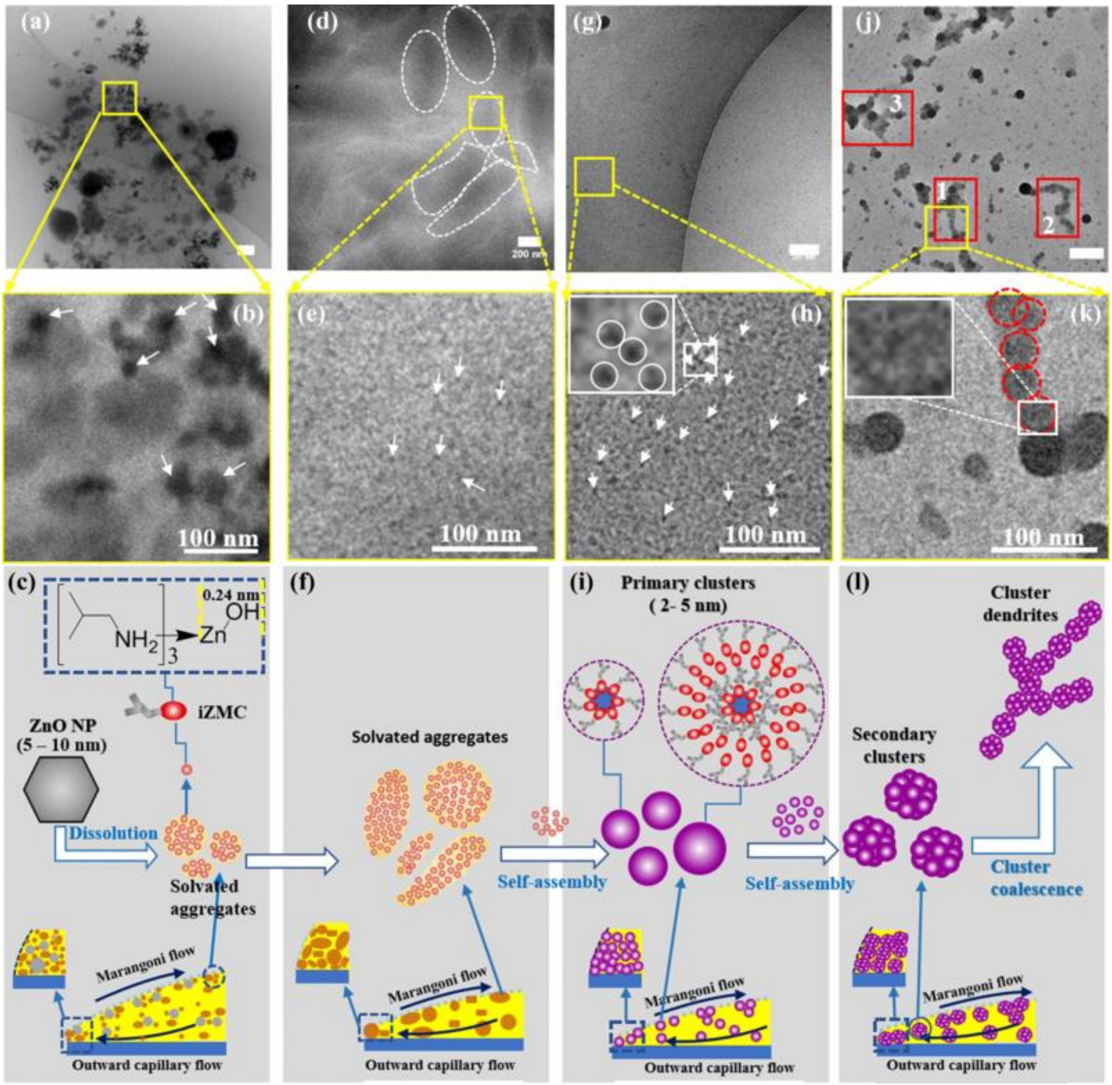

Figure S1. Cryo-TEM images of nano-constituents in an evaporating droplet at different time intervals: 2 min (a), (b), 4 min (d), (e), 6 min (g), (h), and 8 min (j), (k); and corresponding schematic of flows and

cluster formation (c), (f), (i) and (l). Images (b), (e), (h), (k) are enlarged views of the white frames in (a), (d), (g), (j), respectively. The insets in (h), (k) show enlarged views of the white frames in (h), (k), respectively. The scale bars in (a), (d), (g), (j) are 200 nm. The red frames (numbered 1–3) in (k) highlight branched or linear aggregates of coalesced secondary clusters (red dashed circles in (l)). Reprinted with permission from Ref. [2], Copyright (2018) by the American Physical Society.

Cellular patters with dendritic morphologies

As the droplet thins due to the loss of the solvent during evaporation, the dispersion becomes more viscous and Bénard-Marangoni (BM) instabilities are then triggered that coincide with the dendrite growth in the droplet. The induced Bénard-Marangoni flows cause concentration inhomogeneities in a crust composed of the surface active iZMC clusters spread along the drop surface, which in turn results in stress initiating capillary ripples (**Figure S2**a). The solidification of a BM cell is initiated at the centre of the ripple, where the coalescence of the secondary clusters along radial BM flows leads to the branched growth of fibres (**Figure S2**b). These arrested secondary clusters form gel-like residual patterns that compose a three-dimensional swollen fibrillar network. The coalescence of the secondary clusters is believed to be facilitated by inter-cluster solvophobic interactions while the resulting dendritic morphology is attributed to diffusion limited aggregation (DLA) [3]. The difference in the Bénard- Marangoni cell diameters, λ_{BM}, is connected to the local inhomogeneities in the local viscosity and cluster concentration. In a similar manner, cluster coalescence happens upon rapid recession of the contact line, which leads to dendritic growth of swollen fibres forming the peripheral coffee ring (**Figure S2**a(i) and b(i)).

Thanks to the enhanced surface elasticity caused by the surface crust consisting of clusters, the capillary ripples could be observed using video microscopy. The extracted video frames are shown in **Figure S2**c-h which show the cluster pattern formation at sites 1 - 10 and capillary ripples with a constant wavelength $\lambda cp \approx 50$ µm, characteristic for capillary waves in polymer films [4] and soft gels [5]. The estimated viscosity of the fluid layer mediating the ripples was ~20 times larger than the viscosities of the solvents. A similar growth rate of 92 ± 15 µm/s for all the Bénard-Marangoni cells was observed (**Figure S2**i), which is much

higher than the capillary flow rate of *ca* 0.1 μm/s, exhibited by the coffee ring formation [6]. The growth of a single BM cell at site 1 is shown in a set of magnified video microscopy frames in **Figure S2**j-m. Using the relation between the Marangoni number, B, and the droplet thickens, h, [7], the authors calculated B values in order of 300 - 2000, exceeding the critical Marangoni number of $B_C = 80$, above which BM instabilities are induced, *i.e.* the surface tension gradient force is much greater than viscous drag and the rate of heat diffusion [8].

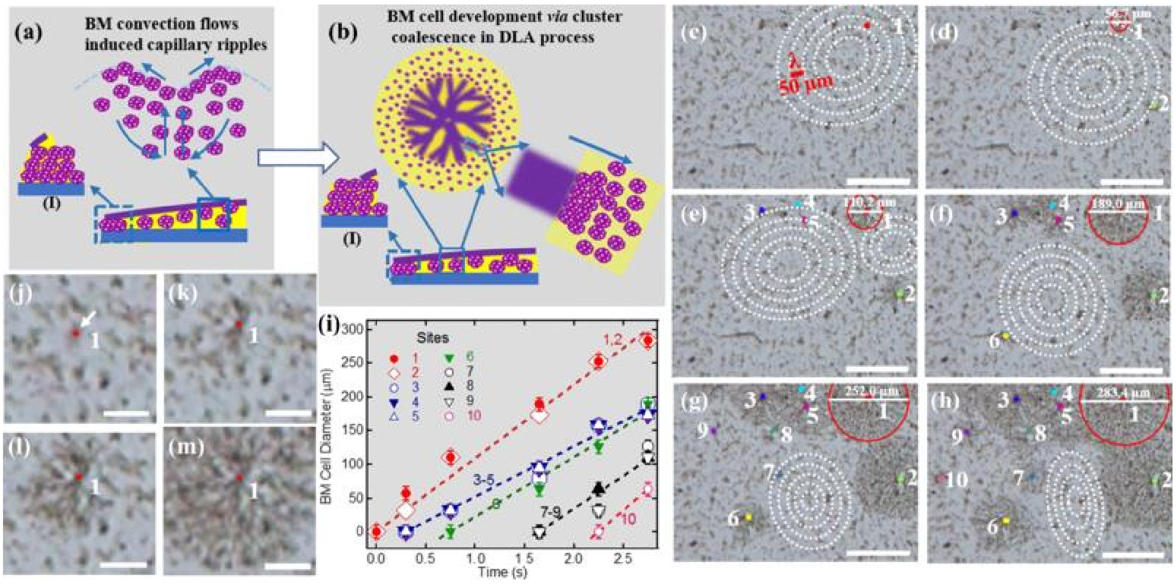

Figure S2. Schematic images of capillary ripples and following BM cell development *via* cluster coalescence (a), (b), and a series of images (c)–(h) extracted from a video at time sequence of 0, 0.3, 0.75, 1.65, 2.25, and 2.7 s respectively: (a) BM convection flows induced capillary ripples (schematically); (b) BM cell formation *via* cluster coalescence in DLA process; (i) the diameter of BM cells at ten site as a function of time, with the growth of the BM cell at site 1 highlighted with red circles from (b) to (f) as an example; and (j)–(m) the magnified images of site 1 in (c), (d), (e), (f), respectively. For the video microscopy experiment, a 100-μL droplet containing 1 mg/mL ZnO nanoparticles (dispersed in a mixture of chloroform/methanol/isobutylamine at ratios of 3.85:1.15:1) was dropped on the glass coverslip (1 mm × 1 mm). The scale bars are 200 μm in (c)–(h) and 50 μm in (j)– (m). Reprinted with permission from Ref. [2], Copyright (2018) by the American Physical Society.

Further evaporation and crystallisation

As the evaporation proceeds, the dendritic morphologies of gel-like residual patterns are preserved, making the constituent fibres well defined. It is believed that the spatial alteration of iZMCs within the clusters is facilitated by solvent molecules, which leads to the formation of multi-layered crystallites with square packing. This self-assembly based, evaporation-driven nucleation in transient aggregates considerably reduces the nucleation barrier, and crystallite formation happens in a near spinodal regime [9], with many crystals forming at once, leading to the polycrystalline structure in the solvated filaments. The ultimate reorganisation of the iZMCs to a lower energy configuration exhibited by multi- layered crystallites with hexagonal packing is subject to the final solvent removal [2].

There is a very interesting hypothesis, suggested by Professor Colin D. Bain, about the role of water molecules in the dissolution of ZnO nanocrystals and subsequent formation of hierarchical residual surface patterns. The moisture assisted dissolution of ZnO has been identified as a key step. Water is characterised by its high permittivity of ~80 at room temperature, which means that water molecules reduce the electric field between charges by the factor of ~80 when compared to the vacuum. Therefore, the presence of water molecules absorbed from air during evaporation of ZnO nanofluid may reduce the energy required for the dissolution to happen. Especially, as it has been shown that relative humidity greater than 45 - 60 % is needed to produce residual surface patterns from ZnO nanofluids without undissolved ZnO crystals [1, 10].

SI.02: Particles

Several different particles were used to prepare nano/microfluids: the in-house synthesised ZnO nanoparticles and commercially sourced ZnO nanopowder (Sigma-Aldrich, <100 nm particle size, ~80% Zn basis), and ZnO powder (Sigma-Aldrich, ACS reagent, ≥99.0% (KT)). Transmission electron microscopy (TEM) images of these particles, taken using JEOL JEM-1400 Transmission Electron Microscope, are shown in **Figure S3**. Particle size distribution and energy-dispersive X-ray spectroscopy analysis of ZnO particles in addition to the synthesis of the in-house ZnO nanoparticles is reported elsewhere [10]. Summarising, particle size distribution was fitted with the log-normal distribution function, thus can be interpreted that *ca* 68.3% of all particles is contained between M/σ_g and $M\cdot\sigma_g$, where M is the

geometric mean particle diameter and σ_g is the geometric standard deviation [11]. The analysis revealed the in-house synthesised ZnO nanoparticles were monodisperse (68.3% diameters between 7.4 and 11.4 nm). Contrary, commercially sourced ZnO nanopowder and ZnO power were polydisperse, with ~68.3% of their maximum size dimension contained in between 36 - 142 nm and 61 - 291 nm, respectively.

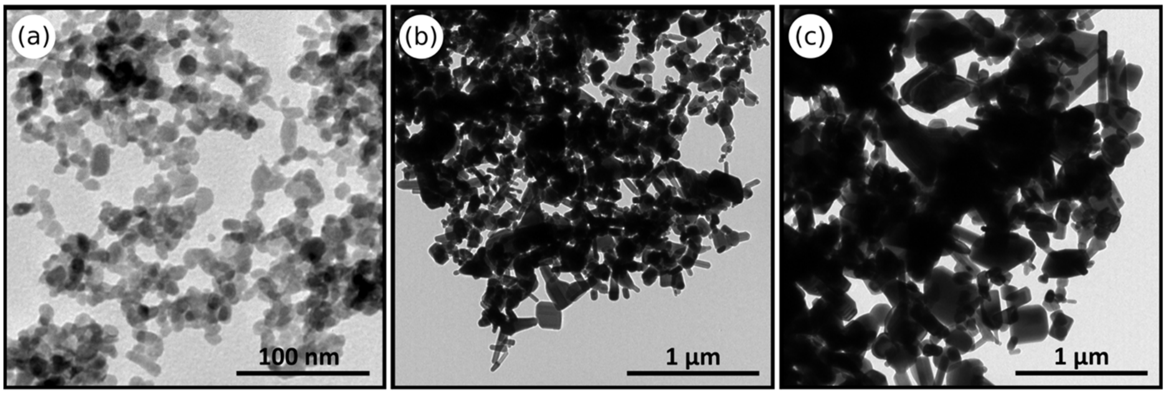

Figure S3. Transmission electron microscopy of different particles used in the study: (**a**) in-house synthesised ZnO nanoparticles, (**b**) ZnO nanopowder, and (**c**) ZnO powder.

X-ray diffraction (XRD) patterns of the particles were taken using the experimental setup described in the main text. A thin layer of each powder was manually deposited onto a bare silicon substrate so that the diffraction image could be recorded. **Figure S4** presents XRD results of the ZnO particles as projections of raw diffraction images into reciprocal space and integrated line profiles. The numbers in parentheses are the Miller indices of the crystallographic planes identified accordingly to the ZnO reference standard (PDF 01-075-0576).

Coherence lengths, L_a, calculated for different peaks of the ZnO particles are listed in **Table S1**. The values were calculated accordingly to the equation $L_a = 2\pi K/\Delta q$, where Δq is the full width at the half maximum (FWHM) of the diffraction peak, obtained by fitting a Gaussian function to the diffraction data shown in **Figure S4**. Their corresponding errors, δL_a, were calculated by the partial derivative method as $\delta L_a = \sqrt{\delta_{\Delta q}^2/\Delta q^2 \cdot L_a^2}$, where $\delta_{\Delta q}$ is error for fitted FWHM Gaussian peak. The calculation of coherence length is described in a great detail in Supplementary Information of Ref. [10]. The average coherence length

error reported in **Table S1** would be better computed as $\overline{\delta L_a} = \sqrt{\delta L_1^2 + \delta L_2^2 + (\dots) + \delta L_n^2}$, or any other appropriate statistical method, however was left as average for simplicity.

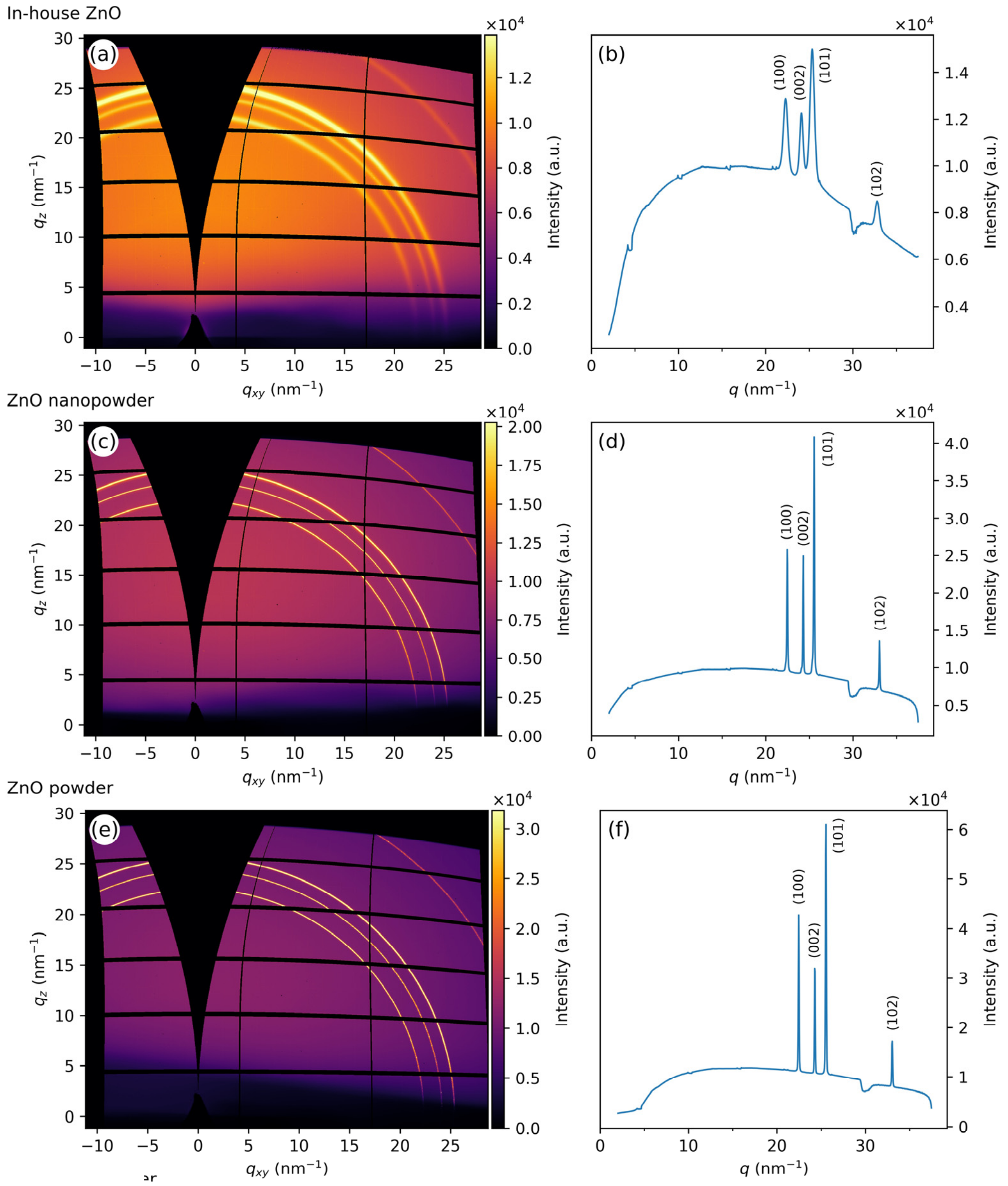

Figure S4. X-ray diffraction data of the particles used in the study: (**a-b**) in-house synthesised ZnO nanoparticles, (**c-d**) ZnO nanopowder, and (**e-f**) ZnO powder. Left-hand side (**a**, **c**, and **e**) shows projections of diffraction images into reciprocal space and right-hand side (**b**, **d**, and **f**) shows integrated line profiles.

Table S1. Calculated coherence lengths (L_a) and errors (δL_a) for the ZnO peaks based on a gaussian fit to the experimental X-ray diffraction data (**Figure S4**) of the in-house synthesised ZnO nanoparticles and commercially acquired ZnO nanopowder and ZnO powder.

	In-house ZnO		ZnO nanopowder		ZnO powder	
Peak (*hkl*)	L_a (nm)	± δL_a (nm)	L_a (nm)	± δL_a (nm)	L_a (nm)	± δL_a (nm)
(100)	11.46	1.04	56.33	5.40	60.73	5.20
(002)	14.81	1.74	67.10	7.69	60.62	7.56
(101)	11.66	0.43	54.89	1.87	55.21	1.87
(102)	12.56	3.53	61.8	17.43	53.10	15.24
average	12.62	2.56	60.03	10.55	57.42	8.33

SI.03: Substrates

Various different substrates cut to 1 cm × 1 cm pieces were used: standard microscope glass slides (type 7101, 0.8 - 1.0 mm thick), silicon wafers (UniversityWafer Inc., ID 452, 100 mm diameter, P type, B dopant, <100>, 0-100 Ω·cm, 500 μm thick, single-sided polish, test grade), both unmodified and hydrophobised by silane functionalisation (see "Silanation of Si wafer procedure" paragraph below), and natural muscovite mica with composition $KAl_2(Si_3Al)O_{10}(OH)_2$ (SJ Trading®, A1 special grade). There has been an effort to use a glass substrate with randomly oriented ZnO nanorods grown on the surface, but it will be described in a separate work. Some of the XRD patterns will be included here for the completeness of the baseline correction description as a surface with crystalline structures was deemed suitable for background calculation algorithm (SI.06). Theoretical information on these substrates is presented in SI.04. All these substrates were scanned using the grazing incidence set-up described in the experimental section in the main text to produce control diffraction data shown below (**Figure S6** and **Figure S7**).

Silanation of Si wafer procedure

Silanation (silanisation) is a process in which long aliphatic chains, such as alkylchlorosilanes, alkylalkoxysilanes and alkylaminosilanes, are grafted on hydroxylated surfaces (*i.e.* silica) *via* trichlorosilane group ($-Si-Cl_3$). As a result, a self-assembled monolayer composed of these aliphatic chains,

driven by the *in-situ* formation of polysiloxane, is anchored to surface silanol groups (Si-OH) *via* Si-O-Si bonds [12, 13]. This is a popular technique used to create (super)hydrophobic surfaces, which is caused by aliphatic chains grafted onto the surface [14]. A schematic representation of this process is shown in **Figure S5**. Initially, the aliphatic chains are attracted to the silica surface *via* the trichlorosilane groups that behave like polar head of amphiphilic molecules. Subsequently, they become hydrolysed when in contact with a water layer adsorbed to the surface. As a result, the aliphatic chains are bonded to the surface silanol groups and to their adjacent neighbours *via* hydrogen bonds. This is followed by a condensation reaction, resulting in a stable and well-oriented network of chains linked to the surface and their neighbours. However, it is suggested that the layer is not linked to the surface by all the individual molecules in as formed network, but by a part of them [12].

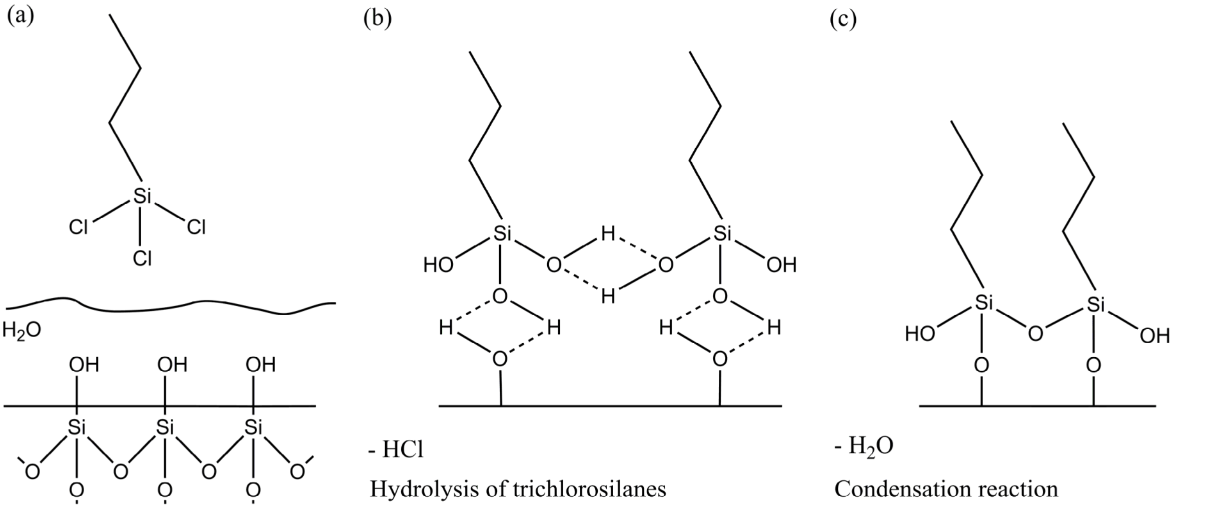

Figure S5. Schematic representation of the silanation process: (**a**) physisorption of trichlorosilane molecule, (**b**) hydrolysis and hydrogen bond formation, and (**c**) water elimination resulting in a chemically bonded monolayer.

In this work, freshly cleaned Si wafer was exposed to UV/ozone for 10 minutes in 42-220 UVO-Cleaner®, Jetlight Company, Inc. Subsequently, the wafer was submerged overnight in a solution of 1*H*,1*H*,2*H*,2*H*-perfluorooctyltriethoxysilane (Sigma-Aldrich, 98%) and dried with a stream of N_2. The wettability of the silanised Si wafer was tested with sessile droplet contact angle (Drop Shape Analyzer

– DSA100, KRÜSS), using *Milli-Q* water at room temperature (~24.5 °C) and relative humidity ~45%. The measured mean water contact angle was equal to 93.7 ± 0.8°.

ZnO nanorods on glass synthesis

Randomly oriented ZnO nanorods on a glass surface were synthesised in a two-step procedure including seeding glass substrates with ZnO nanocrystals [15] and hydrothermal growth of ZnO nanowires [15, 16]. The results from performing evaporation from ZnO nanostructured surfaces will be published separately.

GIXRD control scans of substrates

Control grazing incidence X-ray diffraction patterns of the substrates taken using the experimental setup described in the main text are shown in **Figure S6** for microscope glass slide, unmodified Si, and silanised Si, and in **Figure S7** for muscovite mica and ZnO nanorods on glass. Artefacts which are present in the same positions in all 1D profiles at q equal to ~2.9, 5.3 - 5.9, 12.6 - 13.4, 19.6 - 20.3, 26.0 - 26.7, 31.9 - 32.5, and 36.2 - 37.3 nm^{-1} are produced during the profile integration of 2D diffraction images in reciprocal space likely due to experimental geometry set-up, multimodule detector used (Pilatus 2M, Dectris), and processing pipeline (pyFAI [17] and pyGIX [18]). These integration artefacts are marked with dashed circles in **Figure S6f**. Baseline correction is discussed in detail in SI.06.

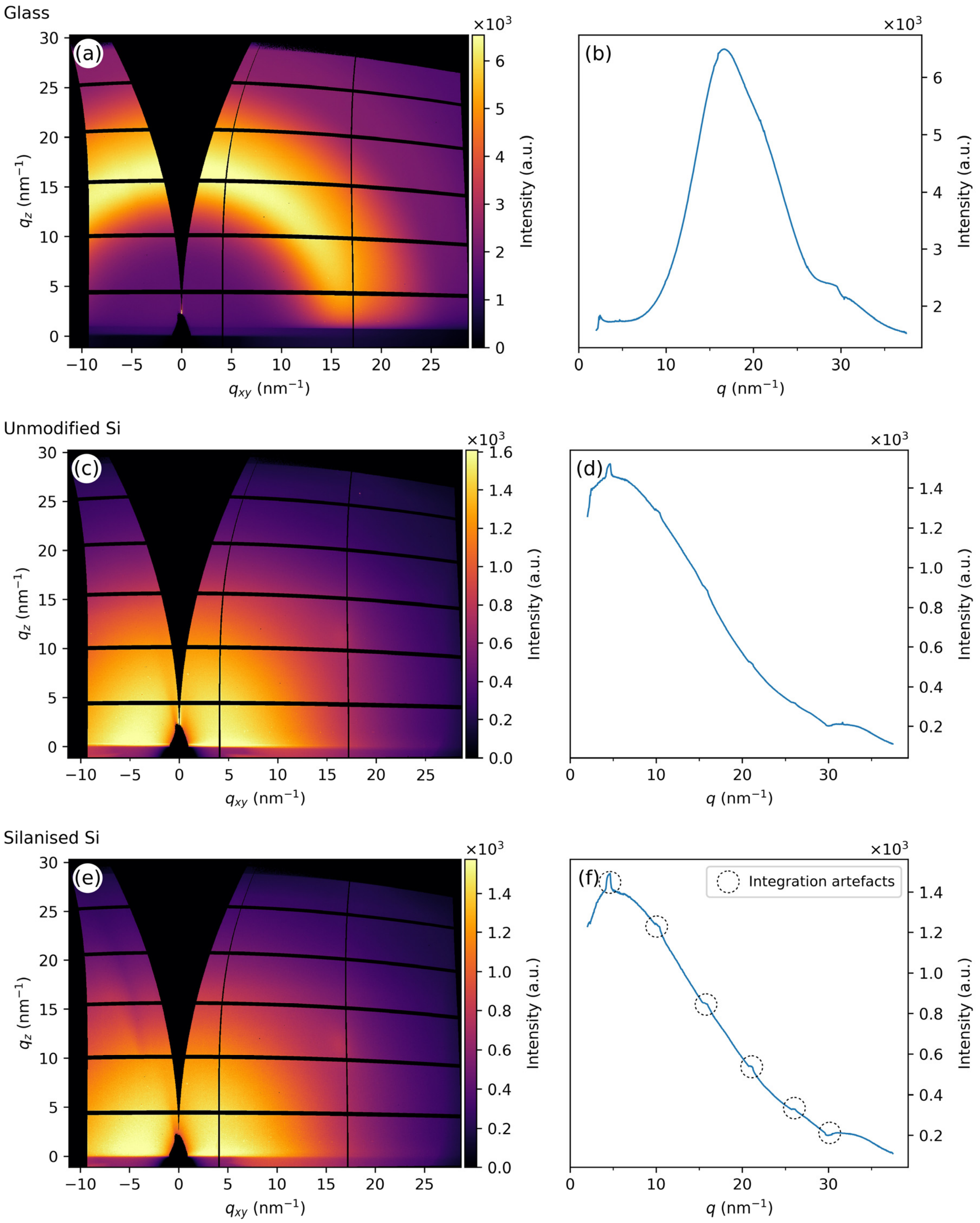

Figure S6. Control grazing incidence X-ray diffraction scans of selected substrates: (**a-b**) microscope glass slide, (**c-d**) unmodified silicon, and (**e-f**) silanised silicon wafer. Integration artefacts in the intensity signal

produced during the profile integration are marked as circles in (**f**). The abbreviation a.u. stands for arbitrary units.

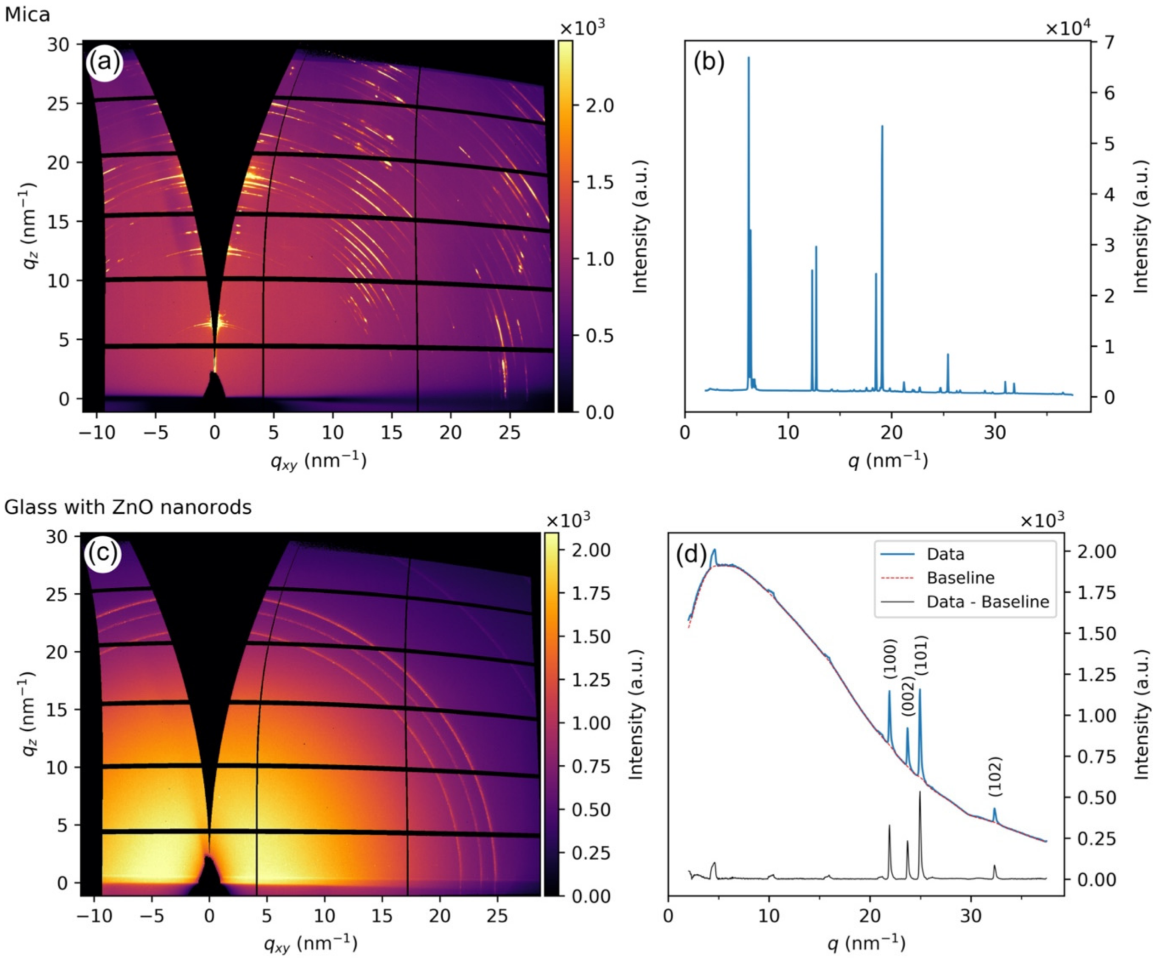

Figure S7. Control grazing incidence X-ray diffraction scans of selected substrates: (**a-b**) muscovite mica, and (**c-d**) glass with grown ZnO nanorods on the surface. Baseline correction (see SI.06) is shown in (**f**).

SI.04: Information about substrates

Microscope Glass Slides

Microscope glass slides are thin flat pieces of glass commonly used for sample preparation, so that the specimen can be held and viewed under a microscope. Due to their low cost, practicable dimensions, and relative ease to cut into appropriate fragments, microscope glass slides can be used as a solid substrate in

a variety of experimental set-ups outside the scope of the optical microscopy. They are usually made of optically transparent glass, such as soda-lime glass (SiO_2 + Na_2O + CaO + Al_2O_3 + fining agents) or borosilicate glass (SiO_2 + B_2O_3 + Na_2O + Al_2O_3 + fining agents) [19].

Tetrahedral SiO_4 units, connected to each other through their corners, are the basic structural elements of silicate glasses and exhibit well-defined geometry. However, the arrangement of these tetrahedral units is characterised by the absence of long-range order in contrast to crystalline silica. Nonetheless, the tetrahedra form a network of ring-like structures that results in ordering on intermediate length scales. The addition of network modifiers such as CaO or Na_2O leads to the generation of non-bridging oxygens. The non-bridging oxygens are connected to the glass network with a single covalent bond and have a negative charge to compensate for the network modifier ions [20]. Schematic silicate glass structure is shown in **Figure S8**.

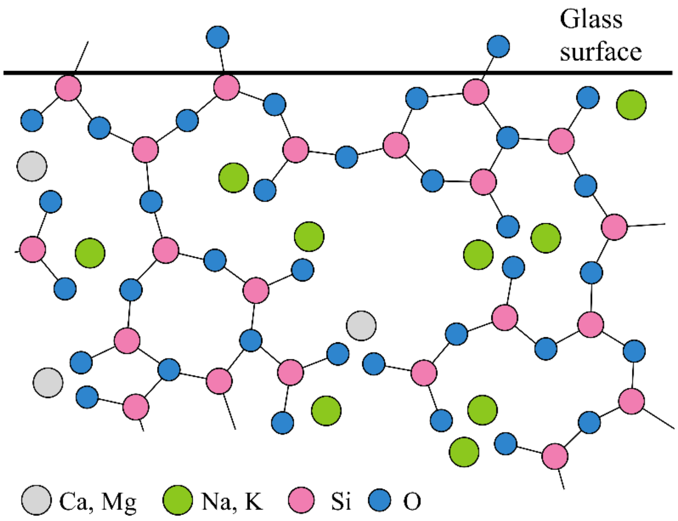

Figure S8. Silicate glass structure. Introduction of network modifier ions such as Ca^{+2}, Mg^{2+}, Na^{+}, or K^{+} leads to creation of non-bridging oxygens. Adapted with permission from Ref. [21]. Copyright (2010) American Chemical Society.

The surface chemistry of the glass is dominated by the hydrous species (Si-OH and H_2O) and surface network structure composed of bridging (Si-O-Si) and non-bridging ($Si-O^-$) oxygens, that have a direct effect on the mechanical strength and chemical reactivity of glass [22]. The interaction with an aqueous

medium often leads to the weathering process and corrosion of the glass surface. The process involves the initial ion exchange of alkaline ions (Na^+, K^+, Ca^{2+}, Mg^{2+}) from the surface for H^+ from hydrogen-bearing species of the medium. This can be followed by the reaction of hydroxyl ions (OH^-) released in the aqueous medium with siloxanes (Si-O-Si) to generate silanol groups (Si-OH) and non-bridging oxygen groups ($Si\text{-}O^-$). The whole process results in a gradual destruction of the surface glass network by appearance of cracks and crystalline corrosion products due to the long term exposure to unfavourable conditions such as gaseous acid pollutants (*i.e.* SO_2 or NO_x) and humidity [21].

Nevertheless, it is expected that the surface chemistry of the microscope slides, made of silicate glasses, is governed by the relative populations of silanol (Si-OH), siloxide ($Si\text{-}O^-$), and siloxane (Si-O-Si) surface groups, similarly to the amorphous silica films [23]. This should be manifested in the water contact angle measurements of microscope glass slides surfaces [24].

Silicon Wafer Substrates

A silicon wafer surface is covered with a native layer of silicon oxide (SiO_2), with a thickness of approximately 0.4 - 2.0 nm, which forms naturally when a silicon surface is exposed to air in ambient conditions [25]. The spontaneous formation of a native oxide at the surface and the ability to grow it further in a control manner make silicon the most used material in the semiconductor industry. A thin layer of SiO_2 acts as a chemically stable protective layer with high quality electrical insulator properties, mainly applicable as a dielectric in numerous devices or a masking layer during device fabrication [26]. The surface of silicon oxide consists of many different chemical species, however the basic chemical moieties present in the structure are silanols (**Figure S9a-c**), siloxanes (**Figure S9d**) and their permutations (**Figure S9e**).

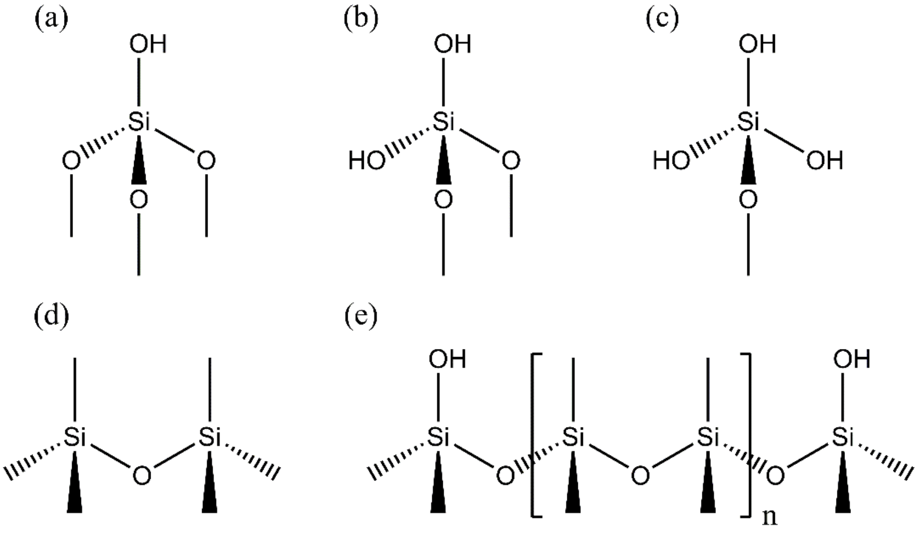

Figure S9. Examples of (**a-c**) silanols, (**d**) siloxanes and (**e**) their permutations present on the surface of a silicon oxide layer. Adopted with permission from Ref. [27]. Copyright (1996) The Electrochemical Society.

The chemical behaviour of the siloxane bridges is determined by the method of the surface preparation, humidity, and thermal and ambient history, particularly for the formation of silanols through hydrolysis. Each chemical moiety existing on the surface will exhibit different properties. This is especially highlighted in their hydrophilicity (water-like nature), susceptible in the water contact angle studies [27]. Silanol groups (Si-OH) present on the SiO_2 surface are responsible for adsorption of water, making it hydrophilic. As shown by Young [28], physical adsorption of water vapour is confined only to the neighbourhood areas of silanol sites on the silica surface, even at high relative pressures. On the other hand, homopolar character of the silicon-oxygen surface bonds (Si-O-Si) prevents water molecules from adsorption on the silica surface, making it hydrophobic. What is more, in addition to siloxanes, researchers have identified other chemical moieties that give the silica surface hydrophobic properties. They can be created as a result of an appropriate silicon wafer (pre)cleaning procedure, *i.e.* with the use of hydrofluoric acid (HF). Amongst these moieties are Si-H, Si-CH_x, and to a lesser extend Si-F [29].

Using different (pre)cleaning/surface modification techniques, it is possible to increase hydrophilic properties of silica by creating more silanol groups (S-OH) on the surface. These include treating silicon

wafers in $HNO_3/H_2O/HF$, HNO_3/HF, or $H_2SO_4/H_2O_2/HF$ solutions followed by rinsing in de-ionized water [30], or exposing wafers to UV/Ozone [31]. Hydrophobicity of silica can be induced by the removal of surface silanol groups by annealing [32] or by grafting of long aliphatic chains on silica surface *via* trichlorosilane group (silanation reaction) [12]. UV/Ozone, thermal annealing and silanation methods are discussed in detail in the following sections.

Mica Substrates

Mica is a group of sheet silicate (phyllosilicate) minerals that exhibit nearly perfect basal cleavage. Muscovite, a representative mineral of the mica family, with formula $KAl_2(Si_3Al)O_{10}(OH)_2$ is often selected as a solid substrate in a range of scientific experiments due to the aforementioned cleavage property. Muscovite mica is built of alternated structures composed of aluminosilicate and K^+ ion layers, parallel to the (001) plane (**Figure S10**) [33]. In the crystal structure, Si^{4+} ions are replaced by Al^{3+} ions at the ratio of 3:1, respectively, making the aluminosilicate layer negatively charged. This negative charge is balanced by the K^+ ion layer that electrostatically binds the two adjacent aluminosilicate layers together. The crystal can be easily cleaved along the K^+ layer between the two $(Si,Al)O_2$ tetrahedron sheets, with K^+ ions usually equally distributed between this two freshly formed surfaces [33].

The aforementioned property is often used to produce an atomically flat, negatively charged and flexible substrate. A freshly cleaved mica displays a hydrophilic nature with a water contact angle equal to zero, and 11% of Si surface atoms constituting silanol groups (Si-OH) [34]. Therefore, when cleaving is performed in air, water molecules adsorb on the surface forming a thin film [33]. Molecular dynamic simulation studies revealed that the adsorbed water molecules form hydrogen bonds with O atoms on the surface, with K^+ ions surrounded by hydrogen bonded water cages [35]. With respect to the ion exchange reaction on the mica surface, two types of adsorption sites were identified concerning permanent and pH dependant charge. The permanent charge type is related to the unbalance structural charge of K^+ ions, and the pH dependant type to protonation or deprotonation of hydroxyl (aluminol and silanol) groups on the tetrahedral or octahedral layers [36].

It is possible to modify the surface of mica by argon/water vapour plasma activation. This results in loss of Al atoms (20-40%) and generation of Si-OH groups (from 11% to 30-32%). Substrates can be further modified by heat treatment that results in the loss of Si-OH and Al-OH groups, and polar Al ions, increasing the hydrophobic character of the surface [34].

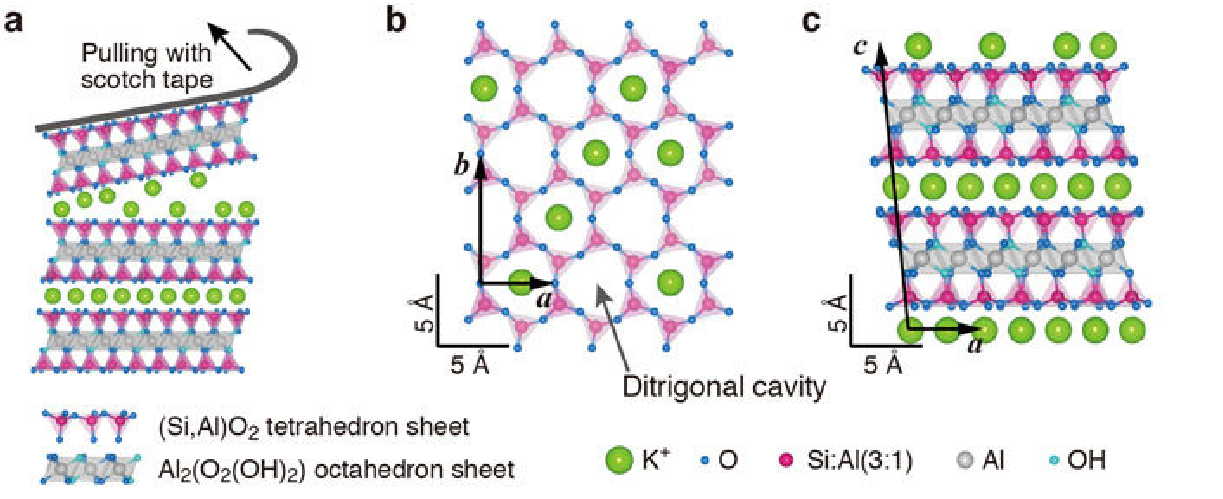

Figure S10. Crystal structure of muscovite mica. (**a**) Cleavage along the K^+ layer between two SiO_2 tetrahedron sheets (along the (001) plane). (**b**) Model of the cleaved (001) surface composed of hexagonal network of $(Si,Al)O_2$ and K^+ ions occupying the ditrigonal cavities of the network. (**c**) Side view of the [010]-directional projection. Si atoms are replaced by Al atoms at the ratio of 3:1, respectively. ***a***, ***b*** and ***c*** are muscovite mica unit cell vectors. Reprinted from [33] under a Creative Commons Attribution 4.0 International License. Copyright 2017 The Authors. No changes were made.

SI.05: Scripts

This section contains examples of scripts used to execute experimental procedures at I07 beamline (Surface and Interface Diffraction) at Diamond Light Source, UK.

time scan

```
pos filterset 0
pos gw_bsy 14

pos hex1rx
pos hex1z
pos temp21
```

```
pos syringe_right

# Tilt sample for GI geometry, change value for different theta
inc hex1rx 0.7

# Dispense nano/microfluid
inc syringe_right - 60

# Scan
cscan testMotor1 0 300 3 pil2roi 0.5 clock temp21 ionc1 1 qbpm3 1

pos filterset 150
```

lateral scan

```
pos filterset 10
pos gw_bsy 14

pos hex1rx
pos hex1z
pos temp21

# Scan 5 mm either side with 0.5 mm step for 2 seconds per step
cscan hex1x 5 0.5 pil2roi 2 clock temp21 ionc1 1 qbpm3 1

pos filterset 150
```

temperature scan

```
# ---Temperature scan that aligns z at each step---

# Define parameters, these can be changed as needed
startTemperature = 50
endTemperature = 400
temperatureStep = 50
annealingTimeInSeconds = 600

# Make sure the full beam intensity is recorded and that the beamstop is in place
pos gw_bsy 14
pos filterset 0

# Make sure to wait for the right temperature
temp21.setAccuracy(1)

# Surface scan along x-axis (2s exposure)
cscan hex1x 5 0.5 pil2roi 2 clock temp 21 ionc1 qbpm3 1

# Temperature loop up
for temperature in range(startTemperature, endTemperature+1, temperatureStep):
        # Set temperature
        pos temp21 temperature
        # z scan to account for thermal expansion
        cscan hex1z 0.3 0.05 pil2roi 1
        # Move to new z value
        cen "roi2_maxval"
        # Do lateral scan across sample at 1 mm increments (1s exposure)
        cscan hex1x 5 1 pil2roi 1 clock temp 21 ionc1 qbpm3 1

# Anneal sample
sleep(annealingTimeInSeconds)
```

```
# Do z-scan again, in case of change
cscan hex1z 0.3 0.05 pil2roi 1
# Move to new z vale
cen "roi2_maxval"
# Do lateral scan across sample at 1 mm increments (1s exposure)
cscan hex1x 5 1 pil2roi 1 clock temp 21 ionc1 qbpm3 1

# Temperature loop down
for temperature in range(startTemperature, endTemperature, temperatureStep):
        # Calculate temperature as ranges only goes up...
        temperature = endTemperature - temperature
        # Set temperature
        pos temp21 temperature
        # z scan to account for thermal expansion
        cscan hex1z 0.3 0.05 pil2roi 1
        # Move to new z value
        cen "roi2_maxval"
        # Do lateral scan across sample at 1 mm increments (1s exposure)
        cscan hex1x 5 1 pil2roi 1 clock temp 21 ionc1 qbpm3 1

# Close shutter and return sample to room temperature and some default beamline settings
pos filterset 150
shclose
temp21.setAccuracy(10)
pos temp21 23
```

SI.06: Baseline correction of diffraction profiles

Baseline correction has been implemented into the data processing pipeline as a baseline trend has been observed in all of the integrated X-ray diffraction profiles (see **Figure S4**, **Figure S6**, and **Figure S7**). This was aimed to increase the contrast and readability of the figures that show the time evolution of the studied processes. We include the details of the baseline correction applied to the GIXRD data in this work for completeness and to familiarise the reader with its outcomes on the data visualisation.

The implemented baseline correction was based on the penalised asymmetric least-squares algorithm (ALS) developed by Eilers and Boelens [37]. In short, a Whittaker smoother is employed to calculate a slowly varying estimate of the baseline. However, positive deviations from the estimated baseline are weighted (much) less than negative ones, which contrasts with ordinary least squares smoothing. This baseline estimation algorithm requires two pre-set parameters that must be tuned to the data at hand. This includes the asymmetry parameter, p, used to calculate asymmetric weights of the residuals (p for positive residual and $1 - p$ for negative residual) and the smoothness parameter, λ. The authors suggested setting the value of p in a range from 0.001 to 0.1 for a signal with positive peaks, and the value of λ between 10^2 and 10^9, subject to exceptions that may occur [37].

Python code implementation of the penalised asymmetric least-squares algorithm was adopted from [38] and the code snipped is shown below. We found that a set of parameters $p = 0.001$ and $\lambda = 10^2$ resulted in a relatively good baseline estimation for a range of diffraction profiles exhibiting sharp peaks. **Figure S11** illustrates results of the baseline calculation algorithm for different values of p (0.001, 0.01, 0.1, and 1) and λ (1, 10^2, 10^4, 10^6, and 10^8) coefficients for an example X-ray diffraction profile data of a glass substrate with grown ZnO nanorods on top of it. Baseline correction performed for the optimal set of p (0.001) and λ (10^2) is shown in **Figure S11e** and highlighted with light green colour.

```
import numpy as np
from scipy import sparse
from scipy.sparse.linalg import spsolve

def baseline_als(y, lam, p, niter=10):
    """
    Calculate baseline

    Baseline is calculated accordingly to the penalised asymmetric least-squares
    (aLS) algorithm (Eilers and Boelens, 2005). There are two parameters for
    asymmetry (p) and smoothness (lambda) that have to be tuned to the data at
    hand. The authors found that generally p between 0.001 and 0.1 is a good
    choice (for a signal with positive peaks) and lambda between 10^2 and 10^9,
    but exceptions may occur.

    Implementation adopted from https://stackoverflow.com/questions/29156532/ by
    Sparrowcide, accessed on 28/08/2018.

    Eilers and Boelens, Baseline correction with asymmetric least squares
    smoothing, 2005.
    https://zanran_storage.s3.amazonaws.com/www.science.uva.nl/ContentPages/
    443199618.pdf, accessed on 28/08/2018.

    Args:
        y (ndarray): data points to calculate the baseline for,
        lam (float): smoothness parameter, generally between 10^2 and 10^9,
        p (float): asymmetry parameter, generally between 0.001 and 0.1,
        niter (int): default 10, the number of iterations.

    Return:
        z (ndarray): baseline calculated for y.
    """

    L = len(y)
    D = sparse.csc_matrix(np.diff(np.eye(L), 2))
    w = np.ones(L)
    for i in range(niter):
        W = sparse.spdiags(w, 0, L, L)
        Z = W + lam * D.dot(D.transpose())
        z = spsolve(Z, w*y)
        w = p * (y > z) + (1-p) * (y < z)
    return z
```

The baseline correction was intended to increase the contrast in the 2D heatmap (waterfall) plots, which show the intensity of the scattered X-rays with respect to the momentum transfer vector, q, and the time of the evaporation process. **Figure S12** visualises the differences between the unprocessed raw data (**Figure S12a-b**), baseline corrected data using the ALS algorithm ($p = 10^{-3}$ and $\lambda = 10^{3}$) (**Figure S12c-d**), and the data corrected by subtraction of a GIXRD line profile of a blank sample scan, *i.e.* bare substrate, measured just before a drop of ZnO nanopowder nano/microfluid was cast on the Si substrate (**Figure S12e-f**), used in this example. The highest contrast in the 2D heatmap was obtained using the ALS algorithm for the base line estimation. However, the relative height of the broad peak related to the droplet on the surface ($q \sim 11 - 14$ nm^{-1}) is significantly reduced when compared to its height in the raw and the blank sample correction data, by a factor of 4 and 2, respectively. This could be improved by increasing the smoothness parameter, λ, however it would reduce the quality of the baseline estimation in the other parts of the line profile. We found the value of $\lambda = 10^{3}$ was a good compromise to the previously established parameters of $p = 0.001$ and $\lambda = 10^{2}$, which performed well for line profiles with sharp peaks.

The baseline correction based on the background subtraction is an attractive alternative as it preserves the relative ratio of the broad peak ($q \sim 11 - 14$ nm^{-1}) to other sharp peaks present in the diffractogram (*e.g.* 22.4, 24.2, and 25.5 nm^{-1} for ZnO) and removes the positive peaks coming from the integration artefacts. However, it amplifies the negative integration artefact peaks, but most importantly, a blank sample scan is not always available for the baseline calculation.

We acknowledge that baseline correction based on the blank sample would be a suitable choice for the example data shown in **Figure S12** in terms of the clarity and readability of the diffraction signal as it reduces the integration artefacts and preserves the relative height of the broad peak to some extent. However, this approach was not always applicable as it required a blank scan taken in the same geometry prior to the measurement the correction is intended for. Therefore, to remain consistent with the processing approach to all data sets, we decided to use the penalised asymmetric least-squares algorithm for the baseline correction as it can be implemented independently and reliably to all diffraction line profiles.

The exact positions of the integration artefacts were determined based on the comparison between the ALS and blank sample corrected diffraction profiles of ZnO nanopowder nano/microfluid dried on a silicon substrate taken as an example, shown in **Figure S13**. It was assumed that the contributions of the integration artefacts due to the detector image processing were the same in the blank sample scans and evaporation data profiles. Therefore, the peaks that only appeared in the raw or ALS corrected data but were not present after the subtraction of the blank sample profile, can be considered as artefacts. **Figure S13a** shows diffraction profiles taken 108 seconds after the start of the drying process (*cf.* **Figure S12**), the top one baseline corrected using the ALS algorithm with parameters $p = 10^{-3}$ and $\lambda = 10^{3}$, and the bottom one with the blank sample scan subtraction. The scattered X-ray intensity 2D heatmaps (q *vs.* time) in **Figure S13b-k**, and sections of diffraction line profiles in **Figure S13l-p** are detailed magnifications of corresponding q ranges marked as blue squares in **Figure S13a**. By comparison of these magnified regions depicted regions in **Figure S13b-p** between the ALS and blank sample corrected profile, it was established that the positive peaks in positions of *ca.* 4.18, 4.68, 15.7 - 16, 20.8 - 21.3, 25.7 - 26.2, 29.3, and 30.9 nm^{-1} and negative peaks in *ca.* 2.7, 5.1, and 29.5 - 30.9 nm^{-1}, are related to azimuthal integration of the detector images (transformation of detector pixels into the reciprocal space coordinates) and considered as the integration artefacts.

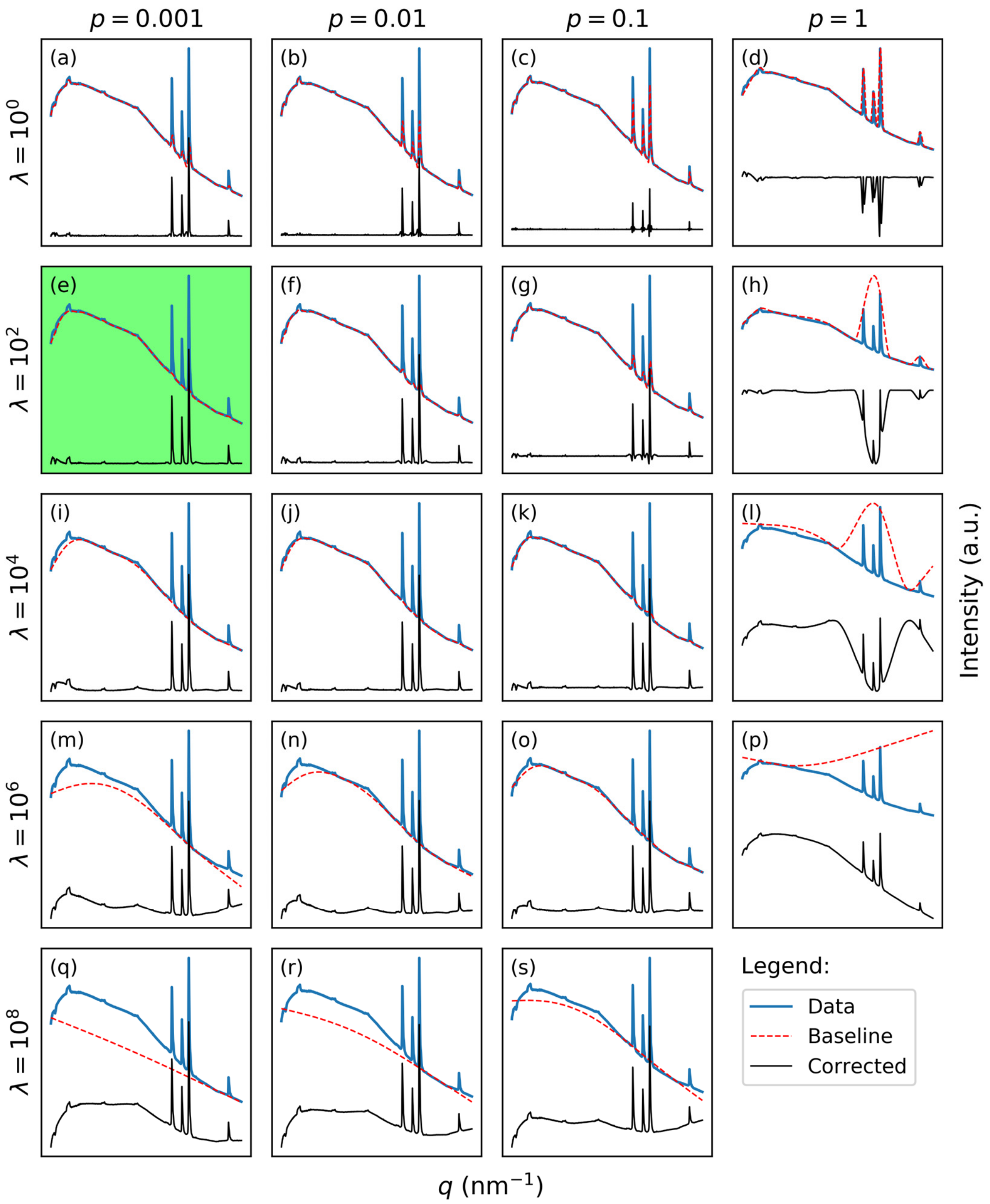

Figure S11. Baseline correction performed for the grazing incidence X-ray diffraction profile of the glass surface with ZnO nanorods grown on top of it (*cf.* **Figure S7**e-f). Baselines were calculated using the penalised asymmetric least-squares algorithm [37] with a different values of asymmetry, *p*, and smoothness, λ, parameters. The set of *p* and λ parameters equal to 0.001 and 100 was found to produce a relatively good baseline estimations for a range of different diffraction profiles. Here, the correction based on this set of *p* and λ is shown in (**e**) and highlighted with light green colour.

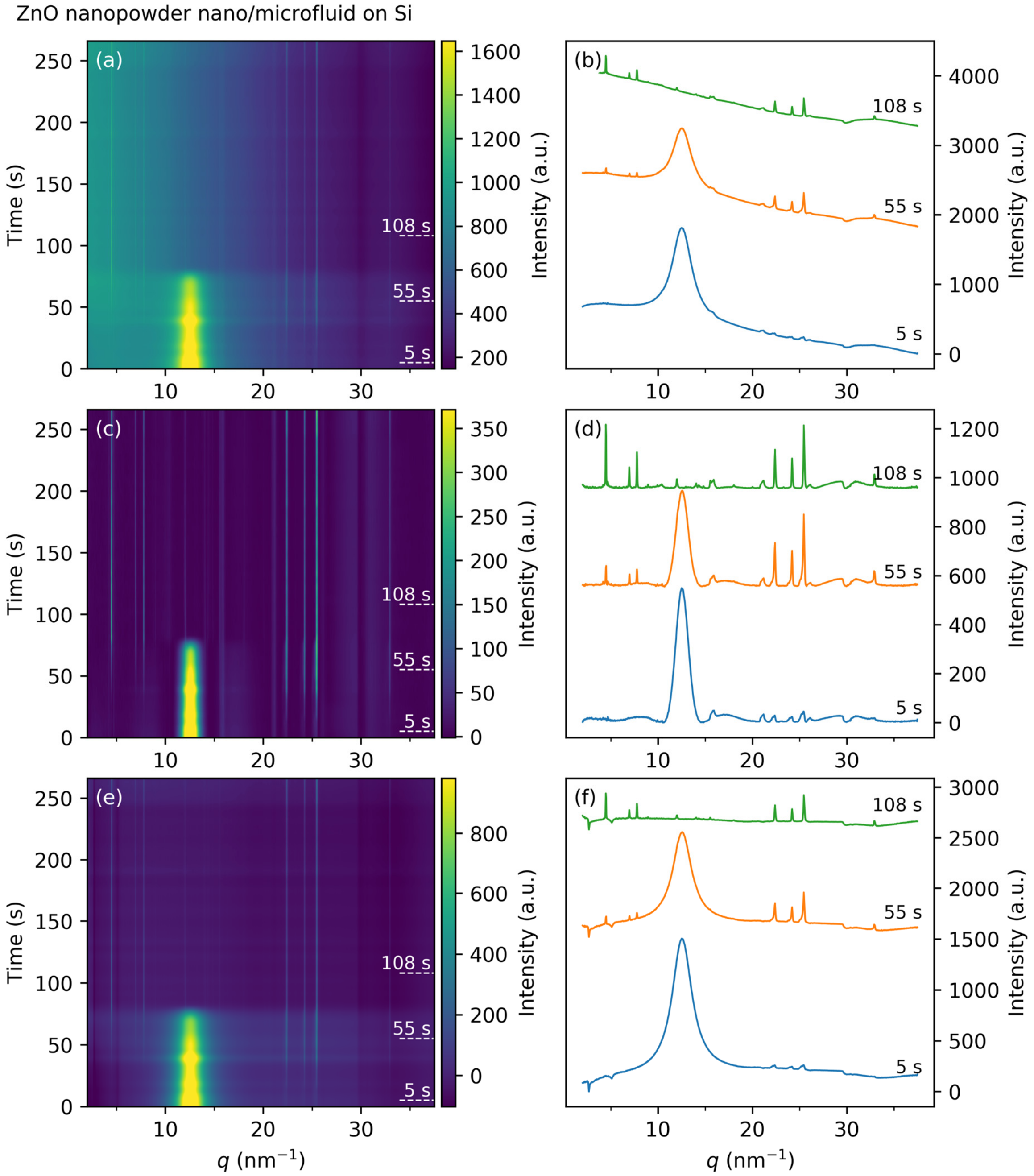

Figure S12. Visualisation of the differences in the 2D heatmaps and 1D line profiles plots with respect to the baseline correction on data produced by a drop of ZnO nanopowder nano/microfluid dried on a silicon substrate. Subplots (**a-b**) show raw data, (**c-d**) data corrected with the penalised asymmetric least-squares algorithm ($p = 10^{-3}$ and $\lambda = 10^{3}$), and (**e-f**) data corrected by subtracting a line profile calculated for a blank scan, *i.e.* bare substrate, taken prior to the deposition of the drop of ZnO nano/microfluid. Dotted lines in

2D heatmaps and lines in 1D profile subplots are labelled with corresponding times counted from the start of the evaporation.

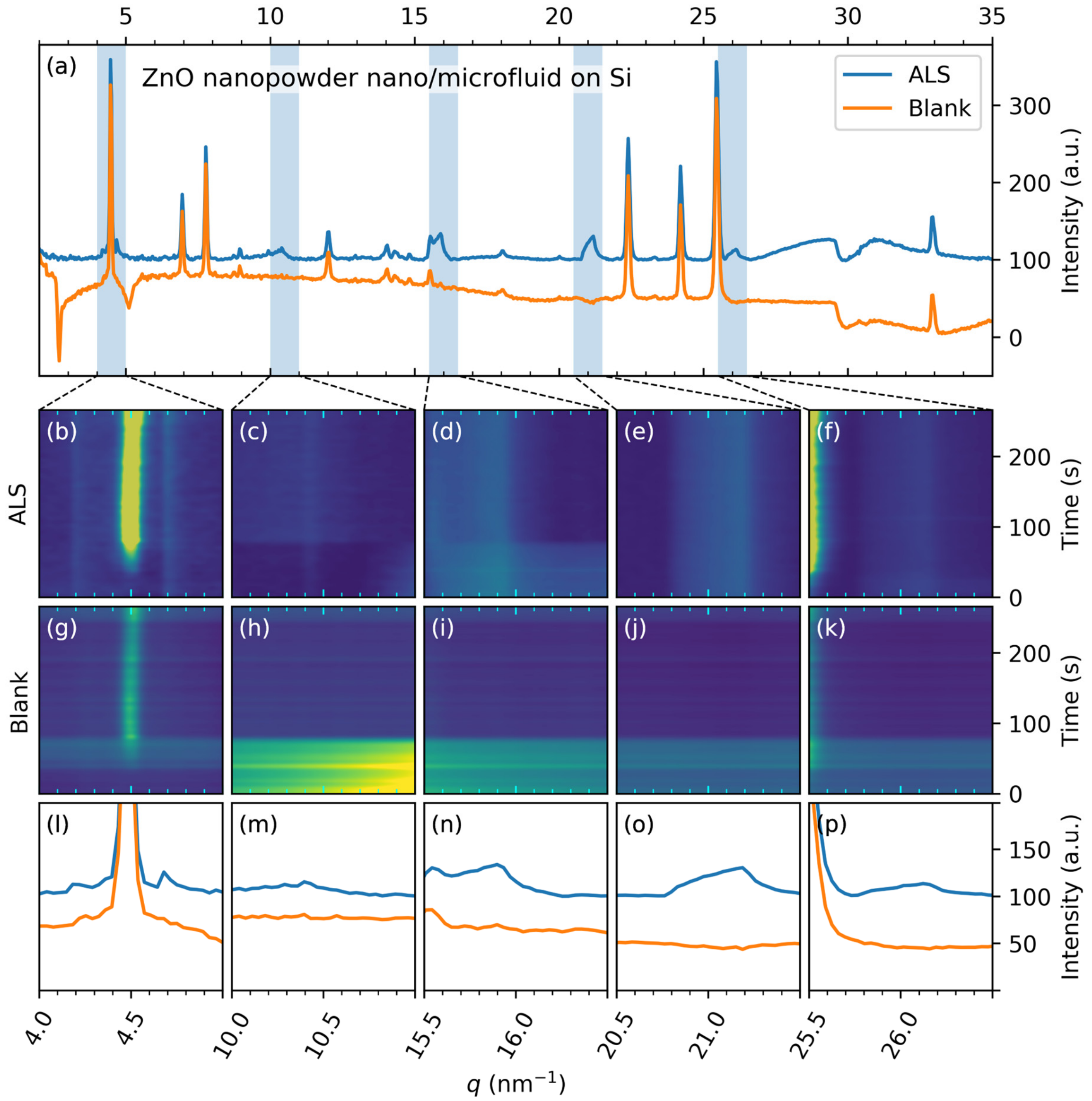

Figure S13. Detailed comparison of two baseline correction methods applied to X-ray diffraction data produced from ZnO nanopowder nano/microfluid dried on Si substrate. (**a**) shows diffraction profiles taken 108 seconds after the start of the drying process, the top one baseline corrected using the penalised asymmetric least-squares algorithm (ALS) with parameters $p = 10^{-3}$ and $\lambda = 10^{3}$, and the bottom one with the blank sample scan subtraction. (**b-f**) and (**g-k**) show the scattered intensity during the evaporation

process for corresponding q ranges marked as blue squares in (a) for the ALS and blank sample methods, respectively. (**l-p**) show diffraction profiles from (a), *i.e.* upper blue for the ALS and lower orange for the blank sample corrections in q ranges corresponding to the marked regions in (a).

SI.07: Time resolved grazing incidence diffractograms

This section contains additional time-resolved GIXRD patterns of ZnO nanopowder and ZnO powder dispersions evaporated on different substrates to investigate the effect of surface chemistry on the evaporation of reactive ZnO nano/microfluids process, similarly to [39], but with the focus on the crystal structure rather than surface pattern morphology.

Droplet of ZnO nanopowder dispersion

Diffractograms recorded for the ZnO nanopowder dispersion evaporated on other substrates such a glass (**Figure S14**), silanised Si (**Figure S15**), and muscovite mica (**Figure S16**).

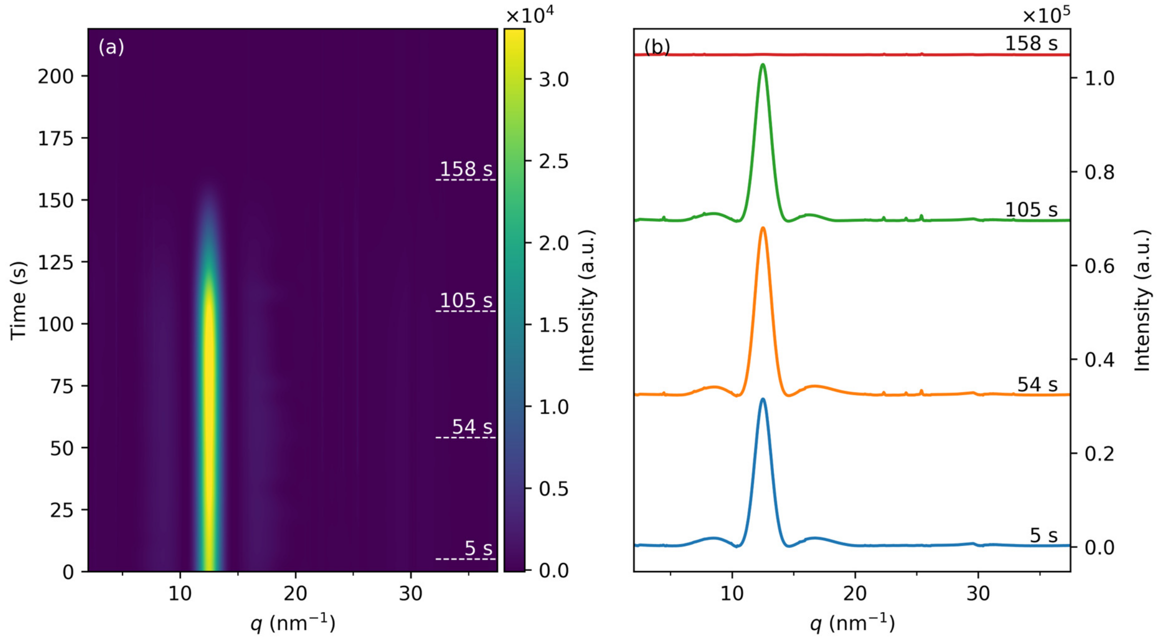

Figure S14. (**a**) Streak plot of the time resolved GIXRD data from a droplet of ZnO nanopowder dispersion drying on glass; (**b**) Snapshots of selected diffraction profiles at different times t = 5, 54, 105, and 158 s of the evaporation process.

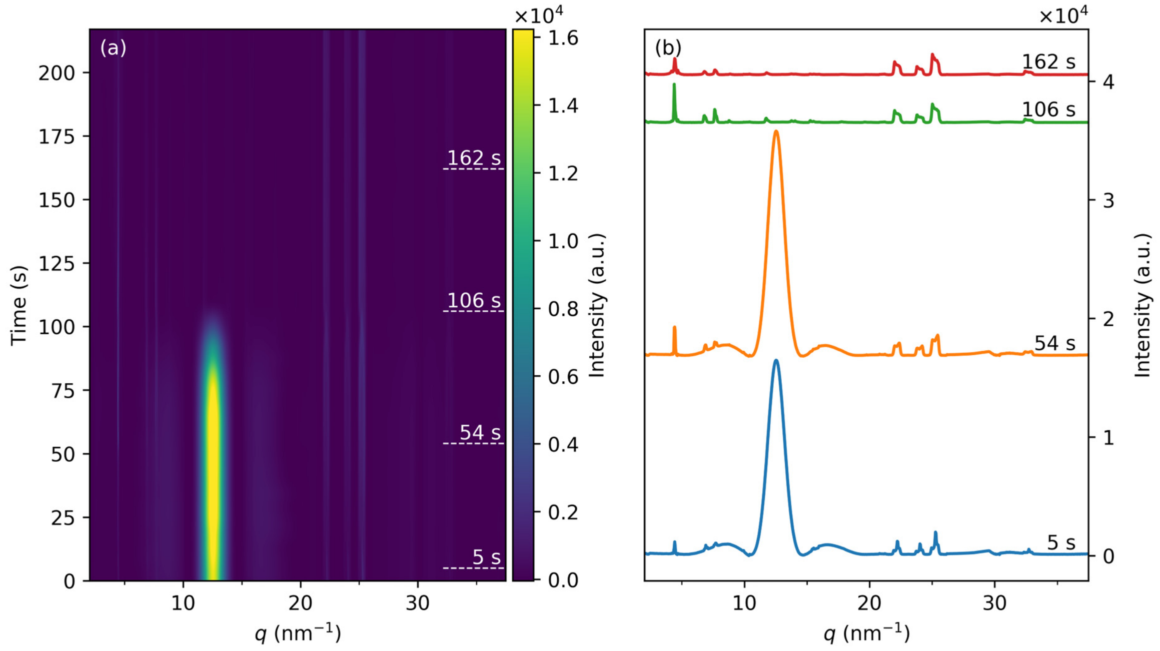

Figure S15. (**a**) Streak plot of the time resolved GIXRD data from a droplet of ZnO nanopowder dispersion drying on silanised Si substrate; (**b**) Snapshots of selected diffraction profiles at different times $t = 5$, 54, 106, and 162 s of the evaporation process.

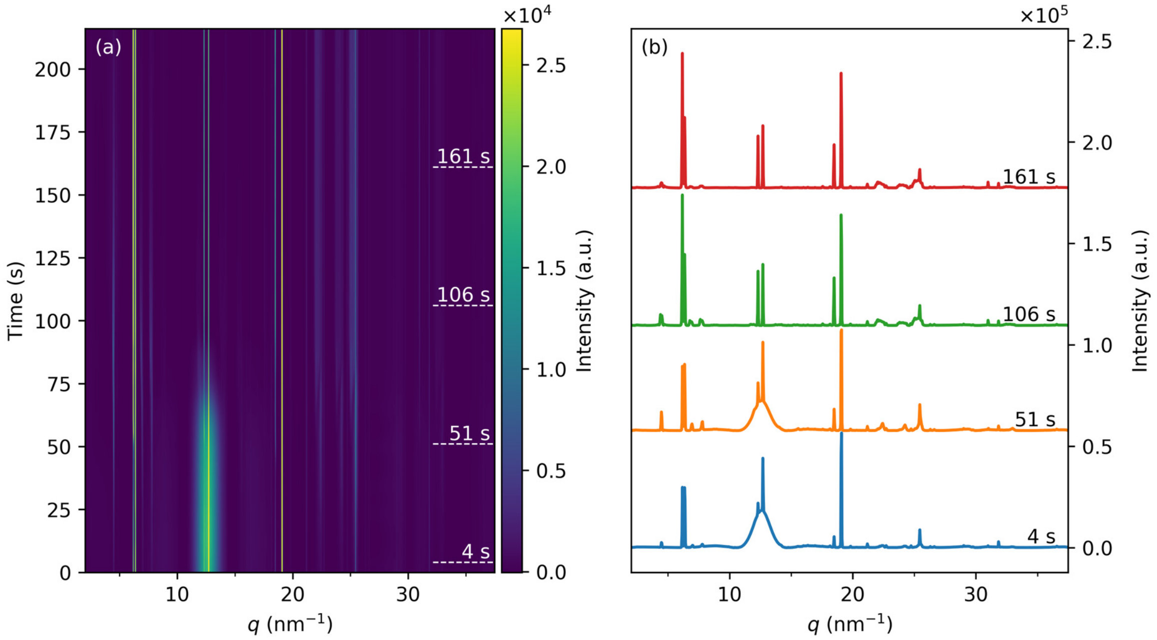

Figure S16. (**a**) Streak plot of the time resolved GIXRD data from a droplet of ZnO nanopowder dispersion drying on mica substrate; (**b**) Snapshots of selected diffraction profiles at different times *t* = 4, 51, 106, and 161 s of the evaporation process.

Droplet of ZnO powder dispersion

Figure S17 shows the time resolved GIXRD data from drying of a droplet containing ZnO powder on a glass substrate. Similar to the ZnO nanopowder droplet (**Figure 7** and **Figure S14** – **Figure S16**), strong diffraction peaks resulting from ZnO crystals at 22.36, 24.21, 25.45, and 32.94 nm^{-1}, indexed respectively as (110), (002), (101), and (102), were present from *t* = 0 s. Their intensities became stronger after *ca* 25 s and reached maximum at *t* ~ 75 s, which coincided with the droplet thinning due to evaporation. The bulk liquid peak at $q = 12.7 \pm 1.0$ nm^{-1} was visible for the first 125 s. A set of three low intensity peaks attributed to LZH structures labelled as 1 (4.50 nm^{-1}), 2 (6.88 nm^{-1}), and 3 (7.77 nm^{-1}) with the first two indexed as (002) and (003) emerged at *t* = 27 s, with a corresponding *d*-spacing of 2.78 ± 0.02 nm. However, their intensity was a fraction of that for ZnO peaks (**Figure 7**b), which is consistent with a slower dissolution rate of the commercially acquired ZnO powder particles due to their size, morphology, and crystallinity [10].

As the drying progressed, other peaks labelled as 4 (8.98 nm^{-1}), 5 (11.89 nm^{-1}), 6 (13.95 nm^{-1}), 7 (15.51 nm^{-1}), and 9 (18.06 nm^{-1}) became more intense and the intensity of peaks 1 – 3 became comparable to those of ZnO (**Figure S17c**), indicating the presence of both layered zinc hydroxides and wurtzite for ZnO. The intensity of the "temporary" peaks around LZH peak 1, labelled as 1a – d at 4.11, 4.68, 5.21, and 5.44 nm^{-1}, respectively, varied throughout the drying process. Peaks 1c-d gradually grew in intensity until the droplet dried at $t \sim 125$ s (**Figure S17c**), then became negligible (**Figure S17d**).

Figure S18 – **Figure S20** show diffractograms collected for the ZnO powder nano/microfluid evaporated on an unmodified Si, silanised Si, and mica substrates, respectively. The diffraction peaks at q = 22.38, 24.21, and 25.45 nm^{-1} appear in every diffractogram since the start of the evaporation and are attributed to the (100), (002), and (101) of the crystallographic planes of ZnO (PDF 01-075-0576). The low q range peaks can be observed in all the diffractograms, indicating the formation of LZH structures, similarly to **Figure S17**.

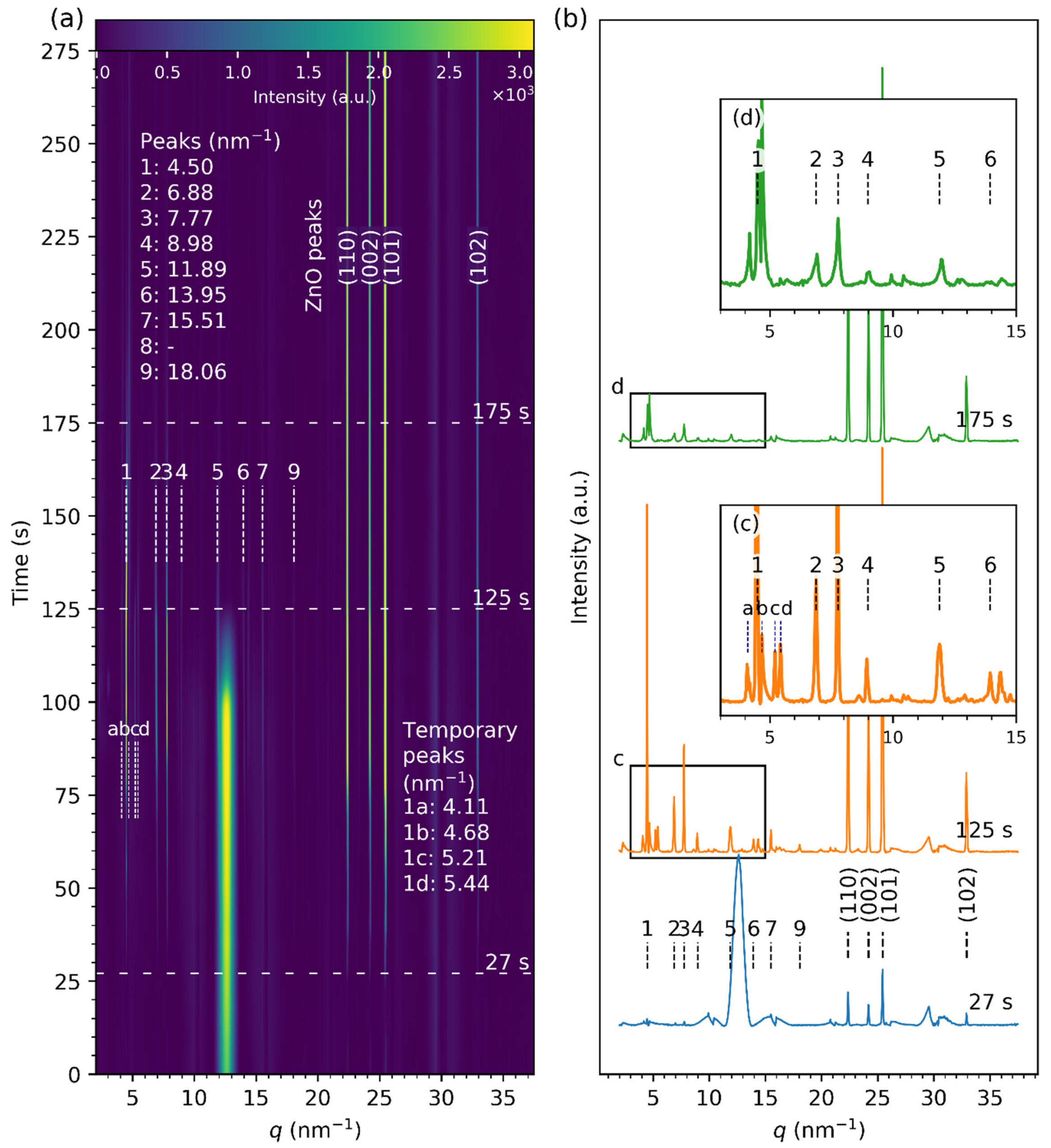

Figure S17. Time resolved GIXRD data for the droplet with ZnO powder on a glass substrate: (**a**) Streak plot of integrated diffraction line profiles with fitted peaks annotated; (**b**) snapshots of selected diffraction profiles measured at different evaporation times; (**c-d**) enlarged view of the diffraction peaks in the low q range.

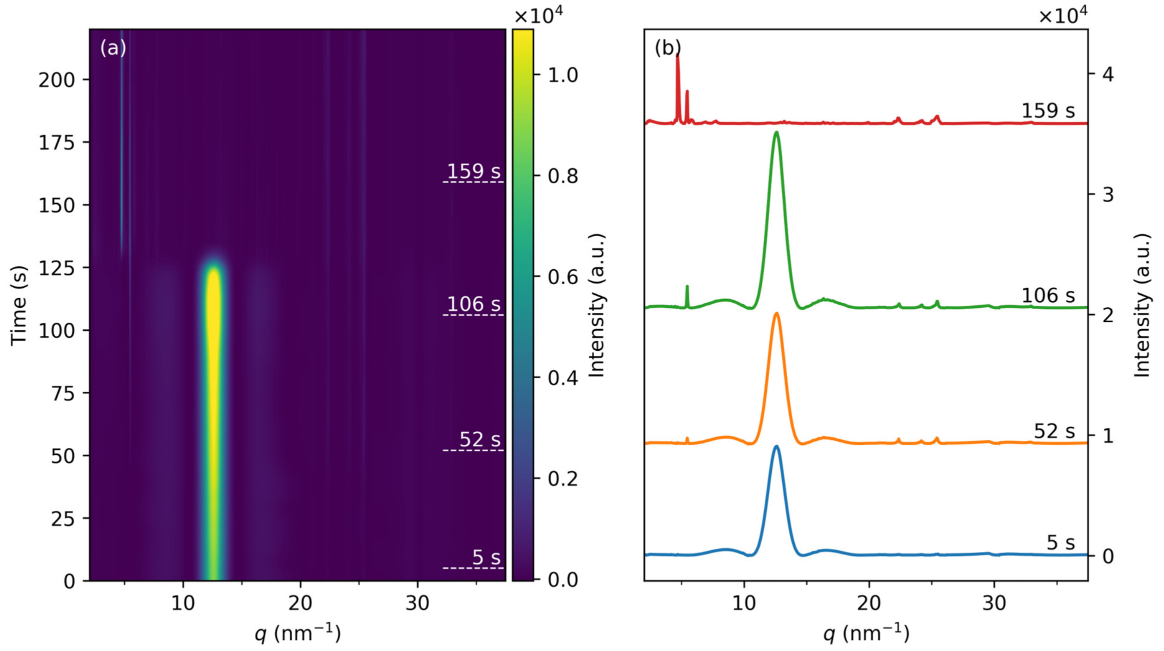

Figure S18. (**a**) Streak plot of the time resolved GIXRD data from a droplet of ZnO powder dispersion drying on unmodified Si substrate; (**b**) Snapshots of selected diffraction profiles at different times t = 5, 52, 106, and 159 s of the evaporation process.

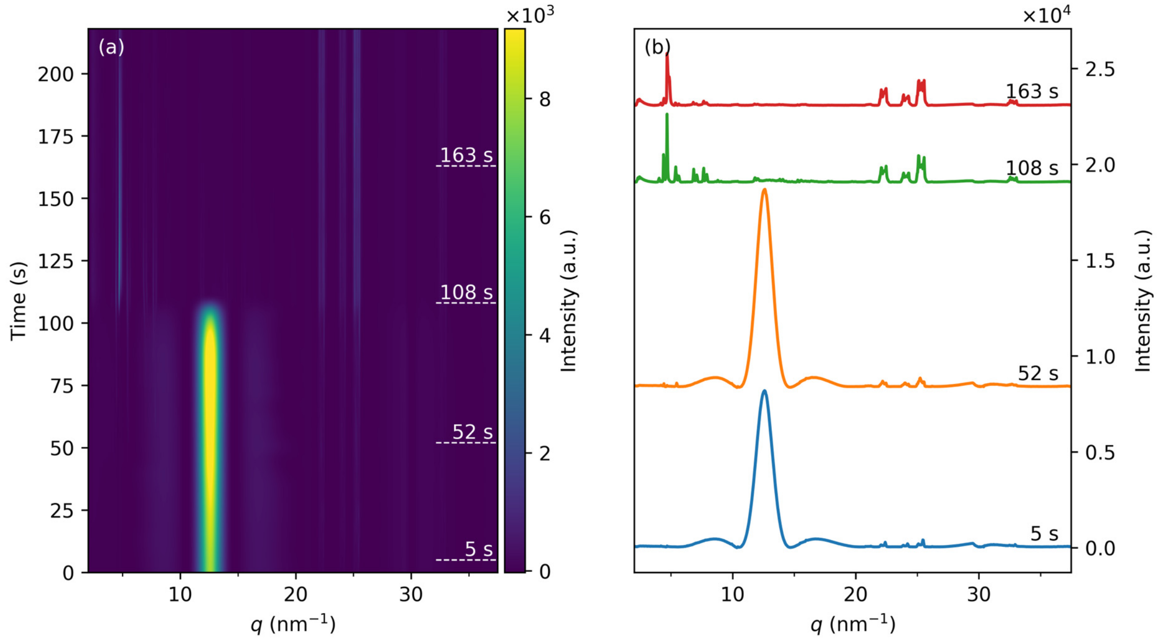

Figure S19. (**a**) Streak plot of the time resolved GIXRD data from a droplet of ZnO powder dispersion drying on silanised Si substrate; (**b**) Snapshots of selected diffraction profiles at different times $t = 4$, 51, 106, and 161 s of the evaporation process.

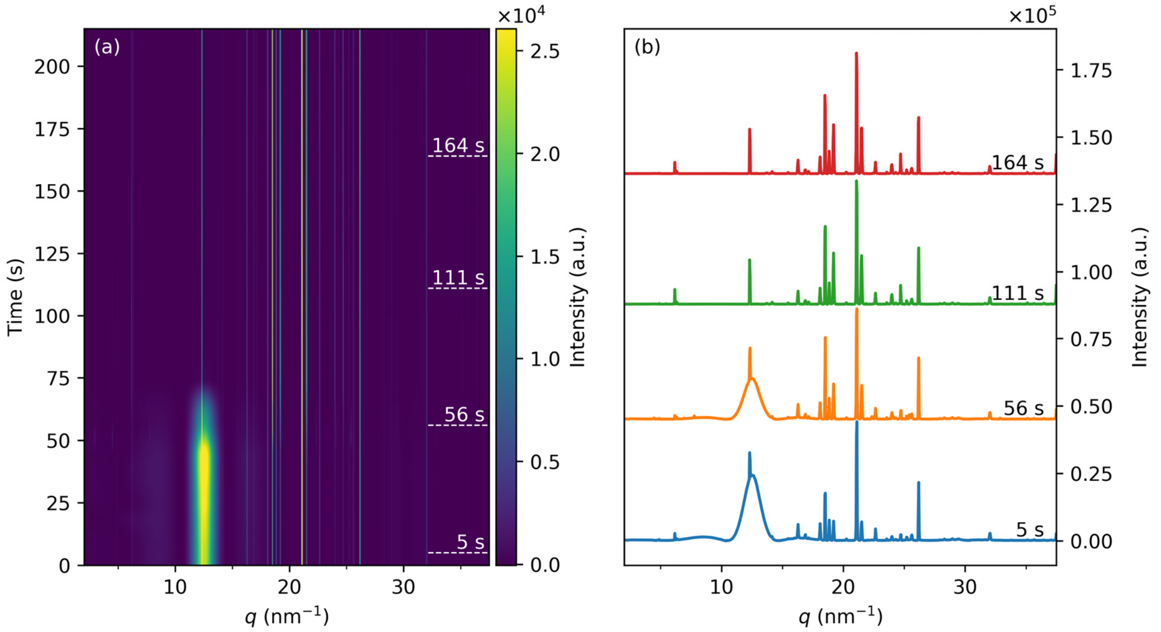

Figure S20. (**a**) Streak plot of the time resolved GIXRD data from a droplet of ZnO powder dispersion drying on mica substrate; (**b**) Snapshots of selected diffraction profiles at different times t = 4, 51, 106, and 161 s of the evaporation process.

SI.08: Coherence length calculations

Coherence length calculations for residual surface patterns X-ray diffraction data follow the same methodology as described in detail in SI.02 above and in SI4 and SI9 sections in Ref. [10]. Coherence lengths, L_a, were calculated for (100), (002), (101), and (102) peaks of ZnO (**Figure S21**) and are listed in **Table S2**.

Table S2. Calculated coherence lengths (L_a) and errors (δL_a) for the ZnO peaks based on X-ray diffraction data for residual surface patterns (**Figure S21**).

	In-house ZnO on silanised Si			ZnO nanopowder on glass			ZnO powder on glass		
Peak (*hkl*)	L_a (nm)	±	δL_a (nm)	L_a (nm)	±	δL_a (nm)	L_a (nm)	±	δL_a (nm)
(100)	8.97		3.46	47.59		10.59	54.00		4.48
(002)	10.77		5.99	47.17		16.38	55.80		7.10

(101)	9.59	2.32	43.33	5.75	49.49	1.72
(102)	9.61	0.20	35.7	2.28	45.98	13.38
average	9.74	2.99	43.45	8.75	51.32	6.67

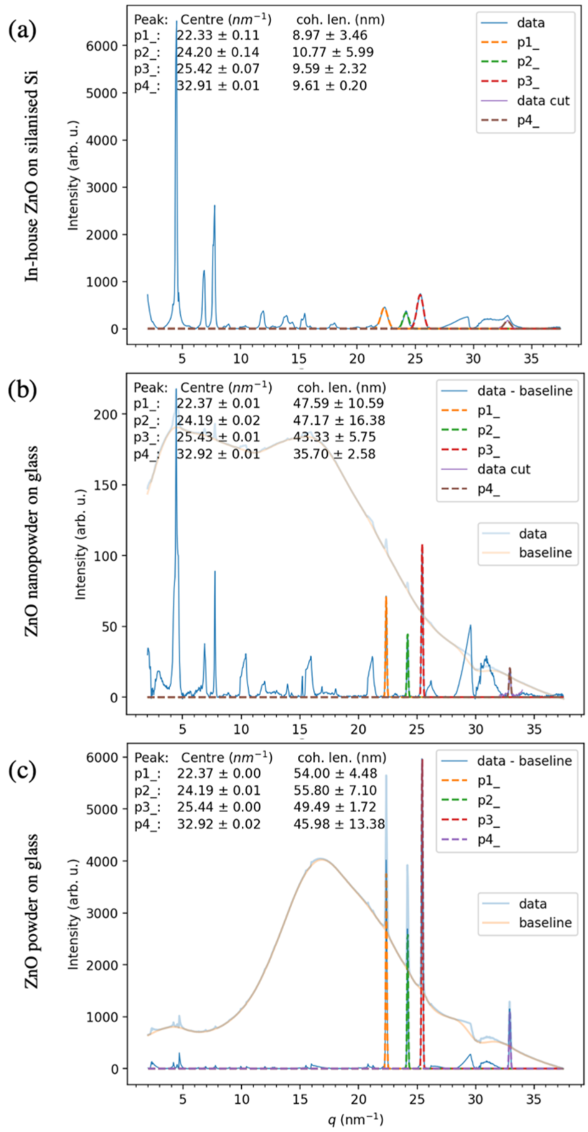

Figure S21. Coherence length calculations for ZnO peaks in residual surface pattern X-ray diffraction data for (a) in-house ZnO dispersion dried on silanised Si, (b) ZnO nanopowder dispersion dried on glass, and (c) ZnO powder dispersion dried on glass.

SI.09: References

(1) Wu, H.; Chen, L. X.; Zeng, X. Q.; Ren, T. H.; Briscoe, W. H. Self-assembly in an evaporating nanofluid droplet: rapid transformation of nanorods into 3D fibre network structures. *Soft Matter* **2014**, *10* (29), 5243-5248, 10.1039/C4SM00887A. DOI: 10.1039/C4SM00887A.

(2) Wu, H.; Briscoe, W. H. Morphogenesis of polycrystalline dendritic patterns from evaporation of a reactive nanofluid sessile drop. *Phys Rev Mater* **2018**, *2* (4), 045601.

(3) Daccord, G.; Lenormand, R. Fractal patterns from chemical dissolution. *Nature* **1987**, *325* (6099), 41-43. DOI: 10.1038/325041a0.

(4) Bandyopadhyay, D.; Singh, G.; Becker, M. L.; Karim, A. Capillary wave confinement-induced stabilization of polymer films. *ACS Appl Mater Interfaces* **2013**, *5* (10), 4006-4010. DOI: 10.1021/am4002502 From NLM.

(5) Kikuchi, H.; Sakai, K.; Takagi, K. Complex propagation of surface waves on soft gels. *Phys Rev B* **1994**, *49* (5), 3061-3065. DOI: 10.1103/PhysRevB.49.3061.

(6) Deegan, R. D.; Bakajin, O.; Dupont, T. F.; Huber, G.; Nagel, S. R.; Witten, T. A. Contact line deposits in an evaporating drop. *Phys Rev E* **2000**, *62* (1), 756-765. DOI: 10.1103/PhysRevE.62.756.

(7) Pearson, J. R. A. On convection cells induced by surface tension. *J Fluid Mech* **1958**, *4* (5), 489-500. DOI: 10.1017/S0022112058000616 From Cambridge University Press Cambridge Core. Barash, L. Y.; Bigioni, T. P.; Vinokur, V. M.; Shchur, L. N. Evaporation and fluid dynamics of a sessile drop of capillary size. *Phys Rev E* **2009**, *79* (4), 046301. DOI: 10.1103/PhysRevE.79.046301.

(8) Maroto, J. A.; Pérez-Muñuzuri, V.; Romero-Cano, M. S. Introductory analysis of Bénard–Marangoni convection. *European J Phys* **2007**, *28* (2), 311-320. DOI: 10.1088/0143-0807/28/2/016.

(9) Filobelo, L. F.; Galkin, O.; Vekilov, P. G. Spinodal for the solution-to-crystal phase transformation. *J Chem Physs* **2005**, *123* (1), 014904. DOI: 10.1063/1.1943413.

(10) Wąsik, P.; Redeker, C.; Dane, T. G.; Seddon, A. M.; Wu, H.; Briscoe, W. H. Hierarchical Surface Patterns upon Evaporation of a ZnO Nanofluid Droplet: Effect of Particle Morphology. *Langmuir* **2018**, *34* (4), 1645-1654. DOI: 10.1021/acs.langmuir.7b03854.

(11) Smith, J. E.; Jordan, M. L. Mathematical and graphical interpretation of the log-normal law for particle size distribution analysis. *J of Colloid Science* **1964**, *19* (6), 549-559. DOI: http://dx.doi.org/10.1016/0095-8522(64)90069-8. Zender, C. *Particle size distributions: theory and application to aerosols, clouds, and soils*. 2013. http://dust.ess.uci.edu/facts/psd/psd.pdf (accessed 2017 12th Dec 2017).

(12) Silberzan, P.; Leger, L.; Ausserre, D.; Benattar, J. J. Silanation of silica surfaces. A new method of constructing pure or mixed monolayers. *Langmuir* **1991**, *7* (8), 1647-1651. DOI: 10.1021/la00056a017.

(13) Ulman, A. Formation and Structure of Self-Assembled Monolayers. *Chem Rev* **1996**, *96* (4), 1533-1554. DOI: 10.1021/cr9502357.

(14) Sriramulu, D.; Reed, E. L.; Annamalai, M.; Venkatesan, T. V.; Valiyaveettil, S. Synthesis and Characterization of Superhydrophobic, Self-cleaning NIR-reflective Silica Nanoparticles. *Sci Rep* **2016**, *6*, 35993, Article. DOI: 10.1038/srep35993

https://www.nature.com/articles/srep35993#supplementary-information. Sun, Z.; Liao, T.; Li, W.; Dou, Y.; Liu, K.; Jiang, L.; Kim, S.-W.; Ho Kim, J.; Xue Dou, S. Fish-scale bio-inspired multifunctional ZnO nanostructures. *NPG Asia Mater* **2015**, *7*, e232, Original Article. DOI: 10.1038/am.2015.133

https://www.nature.com/articles/am2015133#supplementary-information. Lee, E.; Lee, K.-H. Facile fabrication of superhydrophobic surfaces with hierarchical structures. *Sci Rep* **2018**, *8* (1), 4101. DOI: 10.1038/s41598-018-22501-8.

(15) Greene, L. E.; Law, M.; Tan, D. H.; Montano, M.; Goldberger, J.; Somorjai, G.; Yang, P. General Route to Vertical ZnO Nanowire Arrays Using Textured ZnO Seeds. *Nano Lett* **2005**, *5* (7), 1231-1236. DOI: 10.1021/nl050788p.

(16) Law, M.; Greene, L. E.; Johnson, J. C.; Saykally, R.; Yang, P. Nanowire dye-sensitized solar cells. *Nat Mater* **2005**, *4* (6), 455-459, 10.1038/nmat1387. DOI: http://www.nature.com/nmat/journal/v4/n6/suppinfo/nmat1387_S1.html.

(17) Ashiotis, G.; Deschildre, A.; Nawaz, Z.; Wright, J. P.; Karkoulis, D.; Picca, F. E.; Kieffer, J. The fast azimuthal integration Python library: pyFAI. *J Appl Cryst* **2015**, *48* (2), 510-519. DOI: doi:10.1107/S1600576715004306.

(18) Dane, T. G. *pygix - a generic python library for performing reduction of grazing-incidence and fiber X-ray scattering data.* 2016. https://github.com/tgdane/pygix (accessed 06 Jun 2018).

(19) Khatib, J. M. *Sustainability of construction materials / edited by Jamal M. Khatib*; Cambridge : Woodhead ; Boca Raton, Fla. : CRC Press, 2009.

(20) S. Henderson, G.; Calas, G.; F. Stebbins, J. The structure of silicate glasses and melts. *Elements* **2006**, *2*, 269-273.

(21) Melcher, M.; Wiesinger, R.; Schreiner, M. Degradation of Glass Artifacts: Application of Modern Surface Analytical Techniques. *Acc Chem Res* **2010**, *43* (6), 916-926. DOI: 10.1021/ar9002009.

(22) Amma, S.-i.; Luo, J.; Pantano, C. G.; Kim, S. H. Specular reflectance (SR) and attenuated total reflectance (ATR) infrared (IR) spectroscopy of transparent flat glass surfaces: A case study for soda lime float glass. *J Non-Cryst Solids* **2015**, *428*, 189-196. DOI: https://doi.org/10.1016/j.jnoncrysol.2015.08.015.

(23) Warring, S. L.; Beattie, D. A.; McQuillan, A. J. Surficial Siloxane-to-Silanol Interconversion during Room-Temperature Hydration/Dehydration of Amorphous Silica Films Observed by ATR-IR and TIR-Raman Spectroscopy. *Langmuir* **2016**, *32* (6), 1568-1576. DOI: 10.1021/acs.langmuir.5b04506.

(24) Engländer, T.; Wiegel, D.; Naji, L.; Arnold, K. Dehydration of Glass Surfaces Studied by Contact Angle Measurements. *JJ Colloid Interface Sci* **1996**, *179* (2), 635-636. DOI: https://doi.org/10.1006/jcis.1996.0260.

(25) Morita, M.; Ohmi, T.; Hasegawa, E.; Kawakami, M.; Ohwada, M. Growth of native oxide on a silicon surface. *J Appl Phys* **1990**, *68* (3), 1272-1281. DOI: 10.1063/1.347181.

(26) Ghita, R.; Logofatu, C.; Negrila, C.-C.; Ungureanu, F.; Cotirlan, C.; Manea, A.-S.; Lazarescu, M.-F.; Ghica, C. Study of SiO2/Si Interface by Surface Techniques. In *Crystalline Silicon - Properties and Uses*, Basu, S. Ed.; InTech, 2011; p Ch. 02.

(27) Thomas, R. R.; Kaufman, F. B.; Kirleis, J. T.; Belsky, R. A. Wettability of Polished Silicon Oxide Surfaces. *J Electrochem Soc* **1996**, *143* (2), 643-648. DOI: 10.1149/1.1836494.

(28) Young, G. J. Interaction of water vapor with silica surfaces. *J Colloid Sci* **1958**, *13* (1), 67-85. DOI: https://doi.org/10.1016/0095-8522(58)90010-2.

(29) Grundner, M.; Jacob, H. Investigations on hydrophilic and hydrophobic silicon (100) wafer surfaces by X-ray photoelectron and high-resolution electron energy loss-spectroscopy. *Appl Phys A* **1986**, *39* (2), 73-82, journal article. DOI: 10.1007/bf00616822.

(30) Tong, Q. Y.; Gan, Q.; Fountain, G.; Enquist, P.; Scholz, R.; Gösele, U. Fluorine-enhanced low-temperature wafer bonding of native-oxide covered Si wafers. *Appl Phys Lett* **2004**, *85* (17), 3731-3733. DOI: 10.1063/1.1809279 (acccessed 2018/08/04). Gondek, C.; Lippold, M.; Röver, I.; Bohmhammel, K.; Kroke, E. Etching Silicon with HF-H2O2-Based Mixtures: Reactivity Studies and Surface Investigations. *J Phys Chem C* **2014**, *118* (4), 2044-2051. DOI: 10.1021/jp4105757. Ljungberg, K.; Söderbärg, A. Improved direct bonding of Si and SiO2 surfaces by cleaning in H2SO4:H2O2:HF. *Appl Phys Lett* **1995**, *67* (5), 650-652. DOI: 10.1063/1.115191.

(31) Lin, X.; Liao, G.; Tang, Z.; Shi, T. UV surface exposure for low temperature hydrophilic silicon direct bonding. *Microsystem Tech* **2009**, *15* (2), 317-321, journal article. DOI: 10.1007/s00542-008-0703-3.

(32) Lamb, R. N.; Furlong, D. N. Controlled wettability of quartz surfaces. *J Chem Soc, Faraday Trans 1* **1982**, *78* (1), 61-73, 10.1039/F19827800061. DOI: 10.1039/F19827800061.

(33) Arai, T.; Sato, K.; Iida, A.; Tomitori, M. Quasi-stabilized hydration layers on muscovite mica under a thin water film grown from humid air. *Sci Rep* **2017**, *7* (1), 4054. DOI: 10.1038/s41598-017-04376-3.

(34) Liberelle, B.; Banquy, X.; Giasson, S. Stability of Silanols and Grafted Alkylsilane Monolayers on Plasma-Activated Mica Surfaces. *Langmuir* **2008**, *24* (7), 3280-3288. DOI: 10.1021/la703522u.

(35) Odelius, M.; Bernasconi, M.; Parrinello, M. Two Dimensional Ice Adsorbed on Mica Surface. *Phys Rev Lett* **1997**, *78* (14), 2855-2858.

(36) Maslova, M. V.; Gerasimova, L. G.; Forsling, W. Surface Properties of Cleaved Mica. *Colloid J* **2004**, *66* (3), 322-328, journal article. DOI: 10.1023/B:COLL.0000030843.30563.c9.

(37) Eilers, P. H. C.; Boelens, H. F. M. *Baseline Correction with Asymmetric Least Squares Smoothing*. 2005. https://zanran_storage.s3.amazonaws.com/www.science.uva.nl/ContentPages/443199618.pdf (accessed 2018 7 Sep 2018).

(38) @Sparrowcide. *Python baseline correction library*. 2018. https://stackoverflow.com/questions/29156532/ (accessed 28 Aug 2018).

(39) Wąsik, P.; Seddon, A. M.; Wu, H.; Briscoe, W. H. Bénard-Marangoni dendrites upon evaporation of a reactive ZnO nanofluid droplet: Effect of substrate chemistry *Langmuir* **2019**, *35* (17), 5830-5840. DOI: 10.1021/acs.langmuir.9b00109.
